# Overfishing and nutrient pollution interact with temperature to disrupt coral reefs down to microbial scales

**DOI:** 10.1038/ncomms11833

**Published:** 2016-06-07

**Authors:** Jesse R. Zaneveld, Deron E. Burkepile, Andrew A. Shantz, Catharine E. Pritchard, Ryan McMinds, Jérôme P. Payet, Rory Welsh, Adrienne M. S. Correa, Nathan P. Lemoine, Stephanie Rosales, Corinne Fuchs, Jeffrey A. Maynard, Rebecca Vega Thurber

**Affiliations:** 1 Department of Microbiology, Oregon State University, 226 Nash Hall, Corvallis, Oregon 97331, USA; 2 Department of Biological Sciences, Florida International University, 3000 NE 151st St, North Miami, Florida 33181, USA; 3 Department of Ecology, Evolution and Marine Biology, University of California, Santa Barbara, Santa Barbara, California 93106-9610, USA; 4 Department of Ecosystem Sciences, Penn State University, 235 Forest Resources Building, University Park, Pennsylvania 16802, USA; 5 Department of BioSciences, Rice University, 6100 Main Street, Houston, Texas 77005, USA; 6 Department of Biology, University of Florida, Gainesville, Florida 32611, USA; 7 SymbioSeas and Marine Applied Research Center, Wilmington, North Carolina, 28411, USA; 8 Laboratoire d'Excellence «CORAIL» USR 3278 CNRS – EPHE, CRIOBE, Papetoai, Polynésie Française

## Abstract

Losses of corals worldwide emphasize the need to understand what drives reef decline. Stressors such as overfishing and nutrient pollution may reduce resilience of coral reefs by increasing coral–algal competition and reducing coral recruitment, growth and survivorship. Such effects may themselves develop via several mechanisms, including disruption of coral microbiomes. Here we report the results of a 3-year field experiment simulating overfishing and nutrient pollution. These stressors increase turf and macroalgal cover, destabilizing microbiomes, elevating putative pathogen loads, increasing disease more than twofold and increasing mortality up to eightfold. Above-average temperatures exacerbate these effects, further disrupting microbiomes of unhealthy corals and concentrating 80% of mortality in the warmest seasons. Surprisingly, nutrients also increase bacterial opportunism and mortality in corals bitten by parrotfish, turning normal trophic interactions deadly for corals. Thus, overfishing and nutrient pollution impact reefs down to microbial scales, killing corals by sensitizing them to predation, above-average temperatures and bacterial opportunism.

## Abstract (web summary)

Overfishing and nutrient pollution can damage coral reefs in part by increasing coral-algal competition. Here the authors simulate these stressors in a three year field experiment, and show that they interact to enhance sensitivity to temperature, predation and bacterial opportunism.

## Graphical abstract

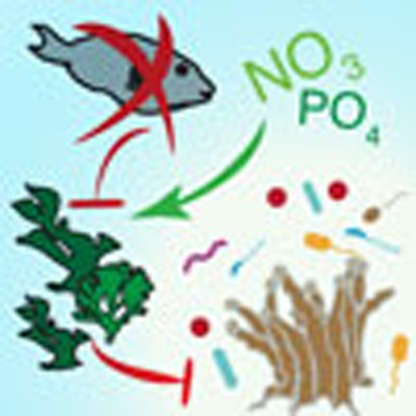

Tropical reefs continue to lose coral cover worldwide due to a variety of anthropogenic stressors, including warming ocean temperatures, nutrient pollution, sedimentation and overfishing^1^,^2^,^3^. Thermal stress from sustained periods of high temperature can kill corals by driving widespread coral bleaching events and coral disease outbreaks^2^,^4^,^5^. Localized stressors can also greatly impact corals. For example, elevated nutrients and overfishing of herbivorous fishes increase algal abundance on reefs^6^,^7^,^8^. Competition with these algae can compromise coral recruitment, growth and survivorship^9^,^10^. The intersecting impacts of these local stressors may ultimately compromise the resilience of reefs to disturbances, such as hurricanes and thermal anomalies^1^,^11^,^12^.

Algal competition and elevated temperatures may also impact reefs by disrupting corals' symbiotic microorganisms, which serve functions ranging from nitrogen fixation^13^ to pathogen inhibition^14^. Decades of coral microbiology research^13^,^14^,^15^,^16^,^17^,^18^,^19^,^20^,^21^,^22^,^23^,^24^,^25^,^26^ demonstrate that coral microbiomes are sensitive to multiple stressors, including algal competition, elevated temperatures and disease^20^,^22^,^23^,^24^,^25^. On the basis of this research, several qualitative and quantitative models have been proposed for the effects of algal competition or temperature on the coral microbiome. The DDAM hypothesis (dissolved organic carbon, disease, algae and microbes)^27^ suggests that turf and macroalgae secrete dissolved organic carbon^28^, which increases growth and oxygen consumption of bacteria^29^,^30^ (but see ref. 31), ultimately harming corals through hypoxia^17^,^26^,^32^. Two additional, non-mutually exclusive hypotheses are that algae produce allelochemicals that directly harm corals, with possible downstream effects on the microbiome^20^ and that interactions with certain algae lead to transfer of algae-associated microbes to corals^19^,^33^.

Sea-surface temperatures also strongly impact the coral microbiome^15^,^16^,^24^,^34^. While mass coral bleaching caused by high levels of thermal stress has received the greatest attention, even modest increases in temperature appear to make corals more vulnerable to opportunistic bacteria. Elevated temperatures increase the release of dimethylsulfoniopropionate from corals. Many opportunistic microorganisms chemotax along gradients of dimethylsulfoniopropionate, potentially allowing them to target thermally stressed corals^34^. Elevated temperatures also increase expression of virulence genes in some opportunistic bacteria^35^, alter innate immune gene expression in corals^36^ and inhibit the protective role of bacteria in the coral mucus^37^. These observations have prompted mathematical models of competition between coral mutualists and pathogens, suggesting that temperature variation mediates changes between pathogen- and mutualist-dominated stable states^25^.

Together, this research strongly suggests that human impacts such as overfishing and nutrient pollution may interact with sea-surface temperatures to cause changes in coral reef benthic communities and their microbiomes that together contribute to coral mortality^17^,^18^,^26^. Much of the foundational work on how corals respond to global and local stressors has been done in short-term, small-scale lab and field experiments based at the organismal level (for example, refs 16, 17, 19, 24, 26). Therefore, it is difficult to extrapolate these prior results to determine how factors that are thought to contribute to coral decline interact to alter coral benthic communities and their microbiomes over ecologically realistic temporal and spatial scales. Therefore, we designed a long-term field experiment to examine how altering herbivory and nutrient pollution impacted coral–algal–microbe interactions within the context of seasonal temperature variation. Specifically, we sought to test the following main hypotheses over ecologically relevant spatial and temporal scales: (1) exclusion of herbivores and nutrient pollution both increase the abundance, and diversity of turf and macroalgae, leading to intensified coral–algal competition and additive increases in coral tissue loss and mortality; (2) coral–algal competition interacts with above-average temperatures to shift coral microbiomes away from their normal configuration and towards distinct, pathogen-dominated stable states; and (3) alterations to the coral microbiome due to increased algal competition or above-average temperatures positively correlate with increased coral disease, and reduced coral growth and survivorship.

To test these hypotheses, we conducted a 3-year field experiment (June 2009–August 2012) that simulated overfishing and nutrient pollution on a reef in the Florida Keys, USA. We tracked their impacts on the benthic community, coral–microbe dynamics and coral survivorship across multiple seasons. To simulate nutrient pollution, four 9-m^2^ plots of reef benthos were enriched in nitrogen and phosphorus, while four control plots remained at ambient nutrient levels (Methods). Enrichment increased nitrogen and phosphorous concentrations approximately four- eightfold above ambient, similar to reefs impacted by nutrient pollution (Methods). We simulated overfishing of herbivorous fishes (for example, parrotfishes and surgeonfishes) by nesting two 1 m^2^ herbivore exclosures and two 1 m^2^ open-topped exclosure controls within each plot (Supplementary Fig. 1). This created factorial treatments of: (1) control, (2) herbivore exclusion, (3) enriched nutrients and (4) herbivore exclusion plus enriched nutrients (Supplementary Notes; Supplementary Data 1 sheet b).

## Results

### Overfishing and nutrients increase algal cover and richness

**Figure 1:**
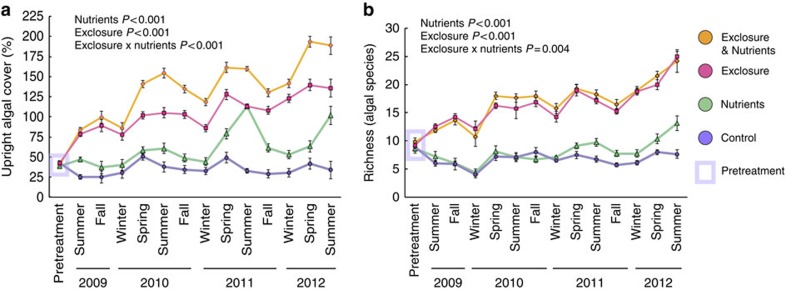
Herbivore exclusion and nutrient pollution alter algal communities. (**a**) Upright algal cover (macroalgae, cyanobacteria and tall turf algae), with herbivore exclusion and/or nutrient pollution over time. Total cover often exceeds 100% due to the three-dimensional algal canopy. (**b**) Macroalgal species richness over time. *P* values are from mixed effect models (Supplementary Table Data 1c). Data are means±s.e.m.

Herbivore exclusion rapidly increased algal cover up to sixfold, species richness up to threefold (Fig. 1a,b) and altered algal community composition (Supplementary Fig. 2; Supplementary Data 1 sheets a–c). Excluding herbivores increased numerous macroalgae (for example, *Dictyota*, *Halimeda* and *Amphiroa*) and tall filamentous turf algae (≥0.5 cm; Supplementary Fig. 2; Supplementary Notes) at the expense of crustose coralline algae and closely cropped turf algae (<0.5 cm), which are beneficial or neutral for coral recruitment, settlement and growth^10^. Nutrient pollution only slightly increased algal richness (Fig. 1b), instead nutrient pollution increased algal cover by driving seasonal blooms of tall filamentous turf algae, *Dictyota* and *Lyngbya* cyanobacteria. Overall, removing herbivorous fishes, and to a lesser extent nutrient pollution, facilitated growth of algae known to increase coral tissue loss or mortality via shading, abrasion and allelopathy^38^,^39^.

### Algae or elevated temperature alter coral microbiomes

To identify how algal communities and nutrient pollution affected the coral microbiome, we collected DNA samples from the surface mucus layer of 80 coral colonies (genera *Porites, Siderastrea* and *Agaricia*) at approximately monthly intervals. From these samples, 478 were used for 16S ribosomal RNA (rRNA) gene amplicon analyses following quality control (Supplementary Data 2 sheets a–d). The phyla Proteobacteria, Cyanobacteria, Bacteroidetes and Actinobacteria were most abundant in all coral genera, with a core set of 13 bacterial orders in ≥95% of all samples (Supplementary Data 3 sheet a). Order Synechococcales (phylum Cyanobacteria), a proposed coral mutualist^13^, was particularly abundant on control corals.

**Figure 2:**
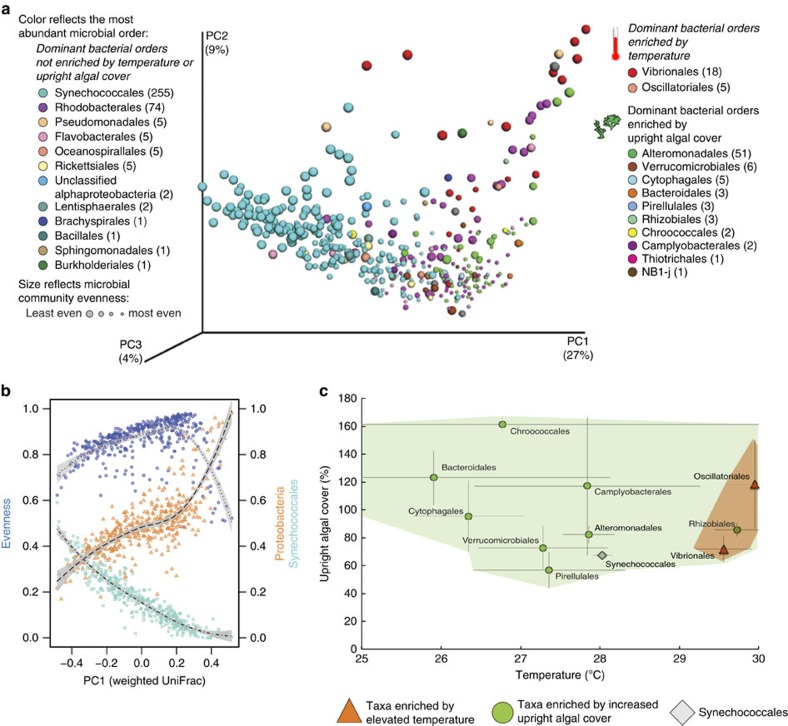
Algal competition and temperature combine to alter coral microbiomes by driving bacterial blooms. (**a**) Principle coordinates analysis plot, summarizing weighted UniFrac distances between coral microbial samples (*n*=435). The main pattern we seek to show here is a shift from *Synechococcus*-dominated communities (cyan) to dominance by a wide variety of other orders as one moves from left to right along PC1. Points are coloured to reflect the most dominant (numerically abundant) microbial order in each sample. Sizes reflect quartiles of microbial community evenness (measured by equitability). Orders that were significantly enriched by temperature or upright algal cover (Pearson regression, FDR *q*<0.05, Supplementary Table Data 3f and 3g) are grouped together in the legend. Parentheses next to each order note the number of samples, in which that order was dominant. Orders that were dominant in ≥15 samples are marked in bold. (**b**) Displacement of Synechococcales by varied Proteobacteria structured differences between stressed coral microbiomes. Points plot the 1st PC axis (**a**) against the relative abundance of Synechococcales (cyan squares, dot-dashed line), Proteobacteria (orange triangles, dashed line) and overall microbial community evenness (blue circles, dotted line). Lines show local regression (Loess regression, span=0.75), with grey bars shading extending to twice the s.e. of the regression. (**c**) Combinations of algal cover (macroalgae, cyanobacteria and tall turf algae) and naturally occurring seawater temperature that allowed algae- or temperature- enriched bacterial orders (**a**) to dominate coral microbiomes. The position of each point, and its associated error bars, represents mean algal cover and temperature, and their s.e.'s, for all samples that were dominated by the labelled bacterial order. Synechococcales, which dominated most (258/435) coral microbiome samples, are included as a reference. Taxa dominating ≥15 samples are marked in bold. Coloured polygons enclose suites of taxa whose mean abundance increased with temperature (orange) or upright algal cover (green; Supplementary Data 3 sheets f–h). Vibrionales and Oscillatoriales responded to both temperature and algal cover, but for Vibrionales only temperature was significant.

Increasing algal cover or elevated temperature suppressed the typical, *Synechococcus*-dominated microbiome of healthy corals and facilitated blooms of other microbes, including many putative opportunists or pathogens (Fig. 2; Supplementary Fig. 3). Alteromonadales, and 10 other orders, many of which were Proteobacteria, increased in abundance as upright algal cover (including tall turf algae, cyanobacteria and macroalgae) increased. Vibrionales and Oscillatoriales increased in abundance with increasing temperature (Fig. 2a; Supplementary Data 3 sheets b–g).

Herbivore exclusion and ensuing coral–algal contact also increased the relative abundance of many otherwise rare microbial orders (Supplementary Table 1), increasing microbial community richness and evenness (Supplementary Data 3 sheet f). In contrast, nutrient pollution suppressed many taxa, allowed fewer bacterial orders to dominate (Supplementary Table 1), and decreased community evenness (Supplementary Data 3 sheet f).

Principle coordinates analysis (PCoA) identified transitions from *Synechococcus*- to Proteobacteria dominance, as the most important axis structuring coral microbiomes (First PCoA axis; Fig. 2a,b). Proteobacteria blooms were accompanied by a drop-off in community evenness (Fig. 2b) and represented 91.9% of bacteria in the least even quartile of samples (Supplementary Fig. 3c). For example, during periods of above-average temperatures, Vibrionales represented up to 62% of the microbial community on individual corals, rising from a mean background level of 5% on unstressed corals.

We used random forest analysis, a machine-learning method, to summarize the extent to which the dominant microbe on coral surfaces could be predicted from external conditions such as algal contact and sea-surface temperature. Random forest analysis predicted displacement of *Synechococcus* by blooms of other microbes with 78.5% accuracy. Temperature and upright algal cover (including both macroalgae and tall turf algae) were the most informative predictor variables (Supplementary Data 3 sheet h). Bacterial opportunists that displaced *Synechococcus* bloomed at different combinations of temperature and overall upright algal cover (Fig. 2c; see Supplementary Data 3 sheet f for turf algae and cyanobacteria specifically). However, conditions favoring different opportunists overlapped, rendering the specific opportunist that displaced Synechococcales partially stochastic and not predictable from random forest analysis of ecological data (Supplementary Data 3 sheet h).

Antibiotic-producing bacteria may play an important role in protecting corals from outbreaks of harmful bacteria^14^. In our data, opportunistic Proteobacteria reached their highest abundance in corals, where Actinobacteria were below ∼2.5% relative abundance (Supplementary Fig. 4), suggesting that antibiotic-secreting Actinobacteria may help to protect corals from opportunists. Actinobacteria, in turn, decreased with increasing algal cover or elevated temperatures, suggesting that these stressors remove an important biotic barrier to potential pathogens (Supplementary Fig. 3; Supplementary Data 3 sheets c,d).

Increasing algal cover or elevated temperatures also shifted the predicted functional profiles of corals towards microbial pathogenesis. We used the PICRUSt software package^40^ to estimate the functional consequences of changes in microbial community composition (Methods). According to these estimates, microbiomes subject to algal competition or above-average temperatures became enriched in pathways involved in opportunism (for example, cell motility and secretion systems) and depleted in pathways for antibiotic production that may help healthy coral microbiomes resist invasion (Supplementary Fig. 5a,b; Supplementary Data 4 sheets a,b). Increasing upright algal cover was also correlated with predicted increases in the abundance of genes for utilization of glycans and pentose sugars in the coral microbiome (Supplementary Fig. 5a). Conversely, upright algal cover was associated with decreases in seven categories of metabolic pathways related to amino acid metabolism and four related to lipid and fatty acid metabolism (Supplementary Fig. 5a). These data support the idea that increased algal cover promotes growth of microbes on the coral surface that are capable of rapid microbial metabolism of algal sugars^17^,^26^,^32^,^41^.

### Algae and temperature increase coral microbiome β-diversity

Stressful conditions may shift coral microbiomes from one stable state to another^25^. We had originally hypothesized that algal competition would produce such a shift in the structure of coral microbiomes. However, we did not find any evidence for such treatment-induced shifts between alternative stable states (Supplementary Fig. 6a). Rather, we found that algal contact and increased ambient temperatures reduced the overall stability of the microbiome as a whole. This type of microbiome destabilization was manifest as increased sample-to-sample variability and quantified using measures of microbiome β-diversity (for example, ref. 42). While β-diversity was originally used to describe species turnover among habitats^43^, in microbial ecology host organisms are often treated as the relevant habitat. This allows β-diversity measures to be used to compare inter-individual variation in the microbiome. This notion has also been generalized to measure species turnover within individuals over time (intra-individual variation). However, few studies (notably ref. 42) have emphasized the relationship between the health of animal hosts and the variability of their microbiomes.

In our experiment, algal contact or elevated temperature drove blooms of microbial opportunists that destabilized coral microbiomes, increasing β-diversity between samples taken from corals subject to these stressors (Supplementary Figs 6a–d and 7a–b). This increased inter-individual and/or temporal variation manifested as increased dispersion of samples from stressed hosts around a centroid of samples from healthy hosts, rather than discrete clusters of samples representing healthy versus diseased hosts (Supplementary Fig. 6).

Algae-driven increases in β-diversity were largely consistent, regardless of whether corals were analysed together or separately by genus (although not individually significant for *Porites*; Supplementary Notes). β-diversity increased in samples where *Synechococcus* was displaced by Proteobacteria (Supplementary Data 3 sheet l), indicating that blooms of Proteobacteria contribute to microbiome instability. Significant microbiome destabilization was also found upon reanalysis of a previous study^19^, in which *Porites* corals were placed in contact with macroalgae (Supplementary Fig. 7c).

### Algal contact induces coral disease and mortality

**Figure 3:**
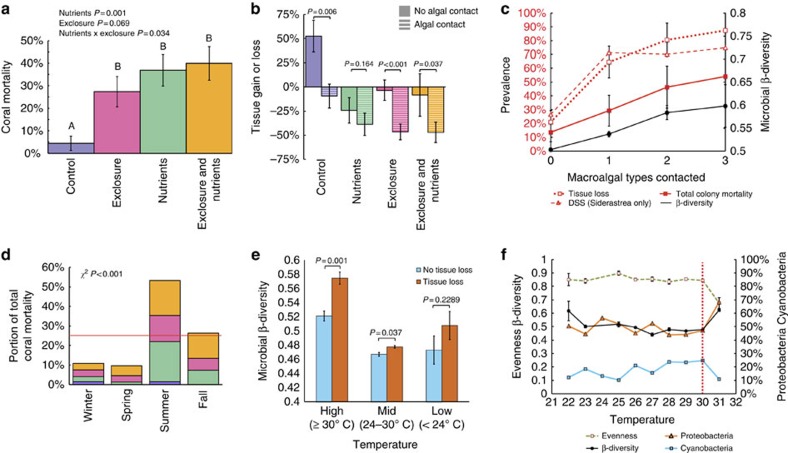
Multiple stressors disrupt coral microbial communities and produce coral mortality. (**a**) Cumulative coral mortality at end of experiment. *P* values are from mixed effect models, letters over bars show differences in Tukey's *post hoc* tests. Herbivore removal significantly increased coral mortality relative to controls (Tukey's *post hoc* test *P*<0.05), but not relative to nutrient pollution alone (*post hoc* test and mixed effects model *P*>0.05). (**b**) Effects of algal contact on coral tissue area, across treatments. *P* values from ANOVAs test the effect of algal contact within each treatment. (**c**) Number of algal taxa contacting corals versus microbiome β-diversity (weighted UniFrac distance), and the prevalence of coral tissue loss, mortality and *Siderastrea* dark spot syndrome (DSS). (**d**) Seasonal distribution of coral mortality, coloured by treatment (**a**). Red line marks null expectation of equal mortality across seasons. *P* value is from a *χ*^2^-test. (**e**) Microbial community β-diversity for corals with or without tissue loss, split by temperature. *P* values reflect non-parametric *t*-tests of distances. (**f**) Temperature effects on coral microbial variability, evenness and relative abundance of Proteobacteria or Cyanobacteria. Evenness and β-diversity data are means±s.e.m. Microbial and coral health data are averaged within each 1 °C interval on the *x* axis. The vertical red line at 30 °C indicates the point nearest to the MMM +1 °C value for our site (30.26 °C); temperatures beyond this result in accumulation of degree heating weeks of coral thermal stress (Methods).

In this long-term experiment, we found that radical changes to the coral microbiome were strongly correlated with coral tissue loss and mortality in the field (Fig. 3). Although corals in control plots grew, gaining 36.8% of initial tissue area on average, other treatments caused 30.6–36.1% tissue loss (Supplementary Data 5 sheets a–d) and six- ninefold increases in mortality (Fig. 3a). Importantly, microbiome evenness was lower and instability (β-diversity) higher (permutational *t*-tests, *P*=0.033 and *P*=0.001, respectively) in corals that lost tissue by the end of the experiment.

Direct algal contact increased coral microbiome instability (Supplementary Fig. 7a), subsequent tissue loss (Fig. 3b, Supplementary Data 5) and mortality (Supplementary Fig. 8a–c). Competition with *Dictyota* algae caused the most severe microbiome disruptions (Supplementary Fig. 7b) and the greatest overall coral mortality (Supplementary Fig. 8c). Further, as the diversity of algal competitors increased, so did microbiome instability, the prevalence of tissue loss and coral mortality (Fig. 3c). In *Siderastrea* corals, algal contact also increased by twofold the prevalence of dark spot syndrome (DSS), a coral disease with poorly understood aetiology (Fig. 3c, Supplementary Fig. 9a). DSS-affected *Siderastrea* had 5-fold greater prevalence of tissue loss and, on average, lost tissue, while *Siderastrea* without DSS had positive growth (Supplementary Fig. 9c,d).

Random Forest analysis predicted coral tissue loss with 76.8±0.11% accuracy using a mixture of benthic and microbial data (Supplementary Data 3 sheet j). Because this analysis compared the susceptibility of colonies to tissue loss rather than the timing of tissue loss, temperature was not included as a variable. The abundance of macroalgae and tall turf algae, which increased coral morality (Supplementary Fig. 8c), were the most important predictors of tissue loss. The next best predictors were the abundance of Rhodobacterales and Rhodospirallales, two putatively opportunistic microbial orders, both in the Proteobacteria and both commonly enriched in diseased corals (for example ref. 44). In this study, Rhodospirallales were enriched by herbivore exclusion (Supplementary Table 1). Together, these data show that the rise of algae following chronic nutrient enrichment and removal of consumer species disrupts coral microbiomes, facilitates disease, increases tissue loss and causes coral death.

### Overfishing or nutrients induce seasonal coral mortality

Surprisingly, coral mortality caused by simulated overfishing and nutrient pollution was strongly seasonal. Although we observed minimal coral bleaching, ∼80% of coral mortality was concentrated in summer and fall (Fig. 3d), which include the warmest months of the year. Yet this seasonal mortality occurred only when herbivores were removed or nutrients increased, suggesting that local stressors and temperature interact to kill corals. If seasonal temperature variation and local stressors do interact to drive coral mortality, we might expect years in which corals suffer greater sub-bleaching thermal stress to correspond to greater coral mortality.

To put treatment outcomes in the context of seasonal temperature variation and coral thermal stress, we calculated raw sea-surface temperatures for our study site using both the Pathfinder v5.2^45^ and HYCOM Gulf of Mexico resources^46^ (Methods; Supplementary Fig. 10). From these we calculated several standard derived measures of coral thermal stress^47^. The maximum monthly mean (MMM) is the average temperature of the warmest month in long-term climate data for our site, excluding the study period (that is, 1982–2009). In predictions of coral bleaching, temperatures that exceed the MMM by ≥1 °C (hereafter MMM +1 °C) are considered ‘thermal stress'^47^. The level of thermal stress is then measured in degree heating weeks (DHWs), which accumulate weekly temperatures above the MMM +1 within a 3-month timeframe (Methods). Because the threshold for increased pathogenesis may be lower than that for coral bleaching^15^, we also calculated DHWs based on the MMM (DHW–MMM) for reference, although the more standard DHW–MMM +1 °C was used in all calculations.

Although summertime temperatures during our experiment exceeded the MMM +1 °C, and positive DHW values accumulated—indicating some thermal stress—in no cases did these values rise to levels that would predict increased bleaching risk (Supplementary Data 6). However, we did observe correlations between sub-bleaching thermal stress and warm-season coral mortality during 2009, 2010, 2011 and 2012, despite limited replication of seasons across years (that is, *n*=4). Across all treatments, the extent of mortality in summer and fall was positively correlated with the extent of sub-bleaching thermal stress in DHWs (*r*=0.960, *P*=0.03), an effect strengthened when control corals were excluded (*r*=0.993, *P*=0.007).

Differences in microbiome stability between healthy and unhealthy corals were exaggerated at temperatures above 30 °C (Fig. 3e), as was the displacement of likely beneficial Cyanobacteria by opportunistic Proteobacteria (Fig. 3f). Regressions using local temperature data (Supplementary Fig. 10b) indicated that both increasing abundance of Proteobacteria relative to Cyanobacteria and reduced microbial community evenness became noticeable around the MMM (29.26 °C) before becoming pronounced around the MMM +1 °C (30.26 °C). This finding suggests that the local MMM may be a critical temperature threshold for the onset of bacterial opportunism.

Temperature variation explained differences in microbial community structure over time better than other measured seasonal parameters (Supplementary Notes). The accumulation of thermal stress due to periods of above-average temperature also appeared to influence microbial communities. Samples in which sub-bleaching thermal stress had accumulated (that is, DHWs>0) showed increased β-diversity (permutational *t*-test on weighted UniFrac distance matrices; *P*=0.001) relative to samples from periods without thermal stress (DHWs=0). Cumulative thermal stress also had small, but significant effects on microbial communities after accounting for daily temperature variation. As the difference in thermal stress between microbiome samples increased, the weighted UniFrac distances between microbial communities also increased (Mantel test, *P*=0.01 and *r*=0.176; Methods), even after accounting for the temperature on the day the sample was taken (partial Mantel test, *P*=0.01 and *r*=0.17). These microbiological changes were probably not due to confounding effects of coral bleaching, as cumulative thermal stress stayed well below the four DHW threshold for mass coral bleaching^5^ (Supplementary Fig. 10a; Supplementary Notes), and little visible bleaching was observed.

### Nutrients alter the outcome of predation on corals

**Figure 4:**
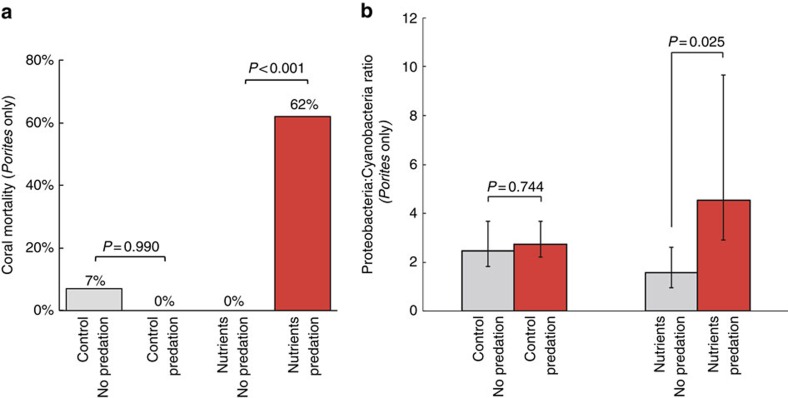
Effects of nutrient pollution and parrotfish predation on coral mortality and microbiology. (**a**) Mortality after predation on *Porites* corals in control or nutrient pollution plots. *P* values reflect Fisher's exact test. (**b**) Effects of predation on the relative abundance of Proteobacteria and Cyanobacteria in *Porites* corals in control or nutrient pollution plots. Parrotfish predation columns (in red) reflect samples taken after the first evidence of parrotfish predation. Error bars in **b** reflect the 95% CI of the ratio. *P* values are from non-parametric *t*-tests.

Coral mortality patterns showed that nutrient pollution changes the impact of important consumers on reefs. Parrotfishes preyed on 63.4% of *Porites* corals in open plots, with a marginally higher predation prevalence in nutrient-enriched plots (*P*=0.08; Supplementary Data 5 sheet f). However, the ultimate outcome of predation for coral health varied greatly under ambient versus nutrient-enriched conditions. Under ambient conditions, the impact of parrotfish predation was negligible, inducing tissue loss in only 7% of bitten *Porites* and never inducing mortality. However, in the presence of nutrient pollution, 92% of *Porites* bitten by parrotfishes lost tissue and 62% eventually died (Fig. 4a). Because other stressors increased potentially opportunistic Proteobacteria compared with Cyanobacteria (primarily Synechococcales), we compared the ratio of these bacterial phyla in bitten corals. The combination of predation and nutrient pollution, but not predation alone, increased the ratio of Proteobacteria relative to Cyanobacteria (Fig. 4b).

## Discussion

We demonstrate that overfishing and nutrient pollution alter benthic communities, causing coral microbiome disruption, blooms of opportunistic coral pathogens and long-term increases in coral disease, tissue loss and mortality. This experimental framework, which combines classical ecological field experiments with microbial time series, allowed us to test key predictions of how coral reefs respond to human impacts over ecologically important time scales and with much greater microbiological detail than has previously been possible in field experiments. The results changed our view of how coral microbiomes respond to environmental stressors; uncovered new interactions among corallivory, nutrient pollution and bacterial opportunism; and quantified how local stressors interact with seasonal temperature variation to impact coral microbiomes and, ultimately, coral survivorship. Together, these results show how altering important trophic interactions can fundamentally reorganize coral reefs down to microbial scales, with multiple negative consequences for reef health.

We predicted that simulated overfishing or nutrient pollution would drive distinct benthic community changes and that these benthic changes would additively increase coral mortality. More specifically, we predicted that exclusion of herbivores would lead to large increases in the abundance and diversity of macroalgae. In contrast, nutrient pollution would only modestly increase macroalgal cover and diversity, instead driving growth of nutrient-limited but unpalatable members of the benthic community such as Cyanobacteria. Similar to past work (for example, refs 6, 7, 8), we show that herbivore removal rapidly leads to over sixfold increases in algal abundance and threefold increases in algal diversity, as well as intense coral–algal competition. Many of the algal genera that increased, such as *Sargassum*, *Dictyota*, *Amphiroa* and *Turbinaria*, cause coral tissue loss or mortality via shading, abrasion and allelopathy^38^,^39^. Nutrient enrichment led to lower overall increases in algal abundance, but facilitated growth of certain taxa such as Cyanobacteria and filamentous turf algae, which can compete intensely with corals^10^. However, the consequences of these local stressors for coral mortality deviated greatly from our prior expectations. We expected herbivore removal and nutrient pollution to have additive effects on corals, with their combined effects causing much greater coral mortality than either individually. Yet, while all treatments significantly increased coral tissue loss and coral mortality above controls, levels of mortality and tissue loss were similar under nutrient pollution, herbivore removal or combined treatments. This pattern likely resulted from different mechanisms of coral tissue loss and mortality operating in the different treatments.

In the nutrient pollution treatment, we traced coral decline to an unexpected interaction among corallivory, nutrient pollution, bacterial opportunism and coral death. Parrotfishes are key herbivores on reefs, but also prey on corals as part of their diet^48^. This corallivory is relatively intense in the Florida Keys, USA where populations of parrotfishes are robust^49^. Here we found that *Porites* corals bitten by parrotfish lost tissue and died at much higher rates under nutrient-enriched conditions than in ambient conditions. In fact, parrotfish corallivory caused no mortality in ambient nutrient conditions. Microbial community shifts towards *Proteobacteria* on the surface of bitten and nutrient-enriched corals (but not bitten corals in ambient nutrient conditions) suggest that increased bacterial opportunism following wounding may cause this increased tissue loss and mortality. It is not clear whether the observed increase in *Proteobacteria* is due to a proliferation of pathogens vectored by parrotfish (as suggested for butterflyfishes^50^) or represents a more general nutrient-driven increase in susceptibility to infection following wounding. In either case, this unexpected observation is especially worrisome, as it shows that nutrient pollution turns parrotfishes, which are normally thought of as coral allies, into agents of mortality for some corals. This finding is important given that nutrient pollution is a problem on many reefs and that restoration of parrotfish populations is an important goal as part of coral conservation and management^51^. Our data suggest that restoration of parrotfishes without efforts to combat water quality issues could have surprisingly negative consequences.

When herbivorous fishes were removed, coral–algal competition and disruption of the coral microbiome appeared to be the main drivers of coral mortality. We showed that algal contact increases coral microbiome richness, facilitates growth of many conditionally rare taxa and increases overall microbiome destabilization. This is broadly consistent with previous reports of algal alteration of coral microbiomes through mechanisms such as provision of algal neutral sugars^41^, secondary effects of alleotoxins on the coral microbiome^20^ and/or transfer of microbes^19^,^33^. In keeping with the prediction that local algal competition will increase disease^27^,^33^ (for example, through dissolved organic carbon^27^ or other mechanisms^33^), we found that the presence of algal competition corresponded to an increased prevalence of DSS^52^ in *Siderastrea* corals. These algae-induced changes to coral microbiology and disease prevalence correlate with increased long-term coral tissue loss and mortality in the field. Thus, our work shows that microbial interactions that were predicted to be important drivers of coral mortality in laboratory studies can be induced by overfishing and nutrient pollution, with important and ecologically meaningful effects for the long-term health of corals.

Our long-term results also provide strong contrasts with existing models of coral microbiome dynamics. Qualitative and quantitative models of the coral microbiome have generally assumed that stress would shift coral microbiomes towards stable states dominated by pathogens (for example, ref. 25). On this basis, we predicted that coral–algal competition, nutrient enrichment and/or above-average temperatures would shift coral microbiomes to alternative stable states. While we did confirm the general idea that multiple stressors increase the abundance of fast-growing opportunists on the coral surface, our data do not support a model in which overfishing and nutrient pollution, ensuing algal competition, or seasonal temperature variation drive coral microbiomes to specific alternative stable states. Instead, we found that above-average temperatures or algal contact destabilized the microbiome, shifting microbial communities from a stable to an unstable configuration. This finding mirrors recent reports in disturbed human and primate microbiomes^42^,^53^, suggesting an underexplored pattern that may be common to many host–microbe systems.

In other animal systems, increased microbiome variability during stress is thought to reflect decreased ability of the host, or its native microbiota, to regulate microbial community composition^42^. Here we show that algal competition and periods of above-average temperature intersect to influence, which bacteria dominate coral microbiomes. Both of these factors promote stochastic blooms of opportunistic bacteria that displace typically dominant members of the coral surface mucus layer. Microbiomes dominated by putatively opportunistic orders, such as Vibrionales, Rhodobacterales, Flavobacterales and Cytophagales were much less stable (as measured by increased β-diversity of microbial communities) than those dominated by Synechococcales. This is interesting because some coral pathogens, such as *Vibrio coralliilyticus* have been shown to suppress coral innate immune pathways^36^. Immune suppression may, in turn, affect the ability of a coral to regulate its microbiome, increasing temporal and/or inter-colony variation in microbiome composition.

Similarly, many reports have quantified the anti-microbial properties of coral mucus^14^, including the contribution of antibiotic-producing Actinobacteria^54^. Our long-term data set suggests that Actinobacteria are important for suppressing opportunists, as outbreaks of Proteobacteria opportunists were more common when Actinobacteria were in low abundance on the coral surface. In agreement with this model, corals exposed to above-average temperature or algal contact showed lower predicted abundances of microbial pathways involved in antibiotic production. Algal contact also reduced the abundance of Actinobacteria, suggesting that algal competition following herbivore removal will lower the natural defenses of corals against potential pathogens, including genera such as *Vibrio* that are especially problematic at high temperatures.

Finally, we find that local overfishing and nutrient pollution interact with seasonal temperature variation to render corals more vulnerable to blooms of harmful bacteria and increased mortality during the warmest months. In contrast, mild, sub-bleaching thermal stress during summer months did not increase coral mortality under control conditions. Connections between local stressors and temperature variation are often discussed in the field in the context of climate change, and have been incorporated into models of reef vulnerability or resilience^55^,^56^, as well as coral disease susceptibility^15^. However, experimental evidence connecting laboratory studies of microbial dynamics to coral mortality in the field has been lacking. In our data, bacterial opportunism increased at temperatures around the local MMM, consistent with past predictions of a threshold at which bacterial pathogenesis becomes especially problematic based on laboratory studies^15^. Also in agreement with previous coral microbiome field studies^16^, we observe blooms of *Vibrio* during periods of above-average temperatures. Motility genes were enriched in these microbiomes, supporting the idea that chemotaxis towards thermally stressed corals, previously shown in microfluidic experiments, may play an important role in coral microbiome dynamics at above-average temperatures^34^. We extend these observations both by documenting how various combinations of algal competition and temperature favour different bacterial opportunists, and linking these blooms to losses of protective symbionts such as *Actinobacteria* caused by local stressors. Finally, our data connects microbiome destabilization caused by these blooms to coral tissue loss in the experiment overall, and especially in periods of high temperature. Thus, multiple lines of evidence collected in this study support an ecologically relevant role for coral microbiomes in mediating coral mortality driven by the intersection of local stressors and seasonal temperature variation.

Together, our results provide experimental data linking prevailing models of how human impacts alter reef ecology^6^ with models of how coral microbiomes respond to algal competition and temperature^15^,^25^. They show that overfishing and nutrient pollution increase the vulnerability of corals to blooms of opportunistic microorganisms and that the impacts of these local stressors are exacerbated by above-average temperatures. Importantly, the coral species that suffered high mortality rates in our experiments are now some of the most abundant on Caribbean reefs^57^,^58^. Thus, some coral species that have withstood the recent decline of more vulnerable relatives may nonetheless be susceptible to increasing local stressors. Clearly, sufficiently extreme thermal anomalies and mass bleaching events will kill corals regardless of local factors. However, our work suggests that conserving natural trophic interactions by protecting herbivorous fishes and reducing nutrient pollution may help stabilize coral microbiomes and shield corals against temperature-driven bacterial opportunism and mortality, at least in the near term^15^.

## Methods

### Experimental design

To simulate the effects of overfishing, nutrient loading or the combination of these stressors, we conducted a 3-year field experiment. Four pairs of 9-m^2^ plots were established. One member of each of these pairs was enriched with nitrogen and phosphorous, while the other remained at ambient nutrient levels (Supplementary Fig. 1). These plots were >10 m from each other in all cases. Each 9-m^2^ plot was delineated into nine 1-m^2^ subplots with metal nails driven into the reef at the corners and centre of each plot. The locations of the plots were selected such that initial variation in rugosity and algal cover within each subplot was minimal. Within each plot, two randomly selected subplots were enclosed with herbivore exclosures, while two other random subplots were selected as exclosure controls. Exclosure controls were fitted with open-topped exclosures. These controls allowed access by herbivorous fishes, but acted as controls for other potential artifacts of the cages.

All exclosures were made of plastic-coated wire mesh with 2.5-cm diameter holes. This diameter mesh generally excludes most fishes >10 cm total length. Smaller or juvenile herbivorous fishes are able to enter the exclosures, but these smaller herbivores generally contribute little to overall grazing rates on reefs and have minimal impacts on the algal communities^59^. In addition, access by smaller herbivores reflects patterns seen under intensive fishing, in which larger fish species are preferentially harvested while leaving smaller size classes of fish^60^,^61^. We scrubbed both exclosures and exclosure controls every 4–6 weeks to remove fouling organisms.

Nutrient pollution was simulated using slow-release fertilizer diffusers applied to each nutrient enrichment plot. Each diffuser was a 15-cm diameter PVC tube, perforated with six 1.5 cm holes. The open ends of the PVC tube were wrapped in fine plastic mesh to keep fertilizer pellets inside, but allow diffusion of soluble nutrients. A total of 175 g of Osmocote (19–6–12, N–P–K) slow-release fertilizer was loaded into each diffuser. PVC enrichment tubes were attached to each metal nail within the 9-m^2^ enrichment plots for a total of 25 enrichment tubes per enrichment plot. Nutrients were replaced every 30–40 days to ensure continued delivery of N and P. Previous studies have shown Osmocote delivery using similar methods to be an effective way of enriching water column nutrients in benthic systems (for example, ref. 62).

### Confirmation of the efficacy of nutrient enrichment

Nitrogen and phosphorus levels were assessed in the water column above each enrichment and control plot as in ref. 52. In July of 2009, 2010 and 2011, divers used 60-ml syringes to slowly draw water from ∼3 cm above the benthos in either control or enriched plots. Samples were taken ∼30 days after nutrient diffuser deployment to ensure that enrichment occurred across the duration of diffuser deployment. Immediately after collection, samples were filtered (GF/F) into acid-washed bottles, placed on ice, returned to the laboratory and frozen until analysed. Dissolved inorganic nitrogen (DIN=ammonium and nitrite+nitrate) and soluble reactive phosphorus concentrations were determined via autoanalyser. We also assessed nutrient enrichment efficiency by analysing tissue carbon:nitrogen (C:N) levels in the common alga *Dictyota menstrualis.* The nutrient content of macroalgae such as *D. menstrualis* reflects ambient nutrient conditions over longer time scales (that is, weeks to months) than ambient water column nutrients (ref. 63 and references therein). We collected *D. menstrualis* from both enriched and control treatments during the same months as water samples. Tissues were dried at 60 °C, ground to a powder and analysed for %C and %N content with a CHN Carlo-Erba elemental analyzer (NA1500). Nutrient data from both water and algal tissue for each replicate were averaged across summers for statistical analysis via analysis of variance (ANOVA).

### Quantification of the herbivorous fish community

Periodically throughout the study, we used 30 × 2 m belt transects (*n*=8) to quantify the density of different herbivorous fishes. Divers slowly swam the length of each transect counting individuals of the different herbivorous fishes in the genera *Sparisoma*, *Scarus*, *Acanthurus* and *Kyphosus*. Fishes were identified to species and their length was estimated to the nearest centimetre. We used published length:weight relationships^64^ to convert fish densities into herbivore biomass. We analysed these data with one-factor ANOVA examining potential differences in herbivore biomass over time.

### Quantification of benthic cover

At least once every season (for example, spring, summer, fall, winter at 12–14 week intervals), we visually quantified benthic cover within four, 50 × 50 cm quadrats in each of the 1 m^2^ treatment areas. These quadrats were divided into 49 points, and benthic organisms under each point were identified to species or genus. Algae that are challenging to identify taxonomically under field conditions (for example, crustose coralline algae and filamentous algae) were classified into algal functional groups. Filamentous algae were classified into short algal turf (<0.5 cm in height) or algal turf (>0.5 cm in height) given that taller, thicker algal turf can often be deleterious to coral health and growth^10^.

Benthic cover was quantified in June 2009 1 week before treatments were initiated to provide a baseline from which to assess changes in algal abundance and community structure. No significant differences among treatments in algal abundance could be detected at the beginning of the experiment (see initial time points in Fig. 1a,b), as expected given random assignment of subplots to treatment conditions. Further, during the summer of each year (2009–2012) when algal cover was often at its highest, we also surveyed open areas of reef (areas that did not have three-sided exclosure controls) within the 9-m^2^ plots to assess whether the exclosure controls had any effect on algal abundance or community composition. We did not detect any differences in algal abundance or community composition between the open unmanipulated areas and exclosure controls (Supplementary Data 1).

### Coral tissue growth or loss analyses

At the beginning of the experiment, we mapped each coral colony in the experimental plots that were >2 cm in diameter and took close-up photographs of these corals *in situ*. Subsequently, we photographed each of these corals every ∼16 weeks throughout the experiment for a photographic record of changes in coral colony health. In each picture a ruler or object of known size was placed next to the coral to provide scale. In total, we tracked the fate of 226 individual corals spread across each of the treatments for over 3 years. The most common corals were *Porites porites* (41.1 % of corals), *Agaricia* spp. (17.7 % of corals), *Siderastrea siderea* (15.5 % of corals) and *P. astreoides* (11.5 % of corals).

These corals allowed us to evaluate the impact of the different treatments on coral growth or tissue loss across the time course of the experiment. We scored growth or tissue loss on a 12-point scale, with bins corresponding to amounts of tissue loss that could be readily observed in photographs (for example, −2=10–25% tissue loss). We scored the tissue loss or gain of each coral over the course of the experiment on the following scale: −6=100% tissue loss, −5=75–90% loss, −4=50–75% loss, −3=25–50% loss, −2=10–25% loss, −1=0–10% loss, 0=0% loss/gain, 1=0–10% gain, 2=10–25% gain, 3=25–50% gain, 4=50–75% gain, 5=75–90% gain and 6>100% gain. We then converted these scores to mean loss/gain by averaging the range corresponding to that score. For example, a coral with a −3 score would be converted to a −37% tissue loss value. Only nine corals grew >100% (score=6) over the course of the experiment. For these corals, we estimated the growth for each coral at 100–500% at 50% intervals (for example, 100, 150, 200% and so on). Statistical analyses were conducted based on the raw tissue gain/loss scores, but converted to percentages in the presentation for ease of interpretation. Further, at each time point we scored each coral for: (1) algal competition as measured by direct contact with algal competitors (and the identification of that algal competitor), (2) the presence of overlying sediment on the coral, (3) predation scars from parrotfishes and invertebrate corallivores (only the former were observed at appreciable levels), and (4) signs of bleaching or disease. The primary coral disease observed was DSS (see ref. 52 for additional discussion).

### Statistical analysis of benthic cover and coral health

Algal cover was analysed using mixed effects models to determine whether the response variable differed among enrichment treatment, herbivore treatment and seasons, as well as whether there was an enrichment × herbivore × season interaction (Supplementary Data 1 sheet c). The nested, split-plot design of the experiment was incorporated into the model by nesting replicates of the exclosures and exclosure controls within ambient or nutrient-enriched plots. We analysed cover for important species or functional groups, as well as for overall upright algal cover, which is a proxy for the competitive environment of corals. Upright algal cover included all macroalgae and tall filamentous turf, but excluded crustose coralline algae and short filamentous turf as these two functional groups are relatively benign for corals^10^. In addition, we assessed how herbivore exclusion, nutrient pollution and season impacted algal community structure via PCoA of Bray–Curtis divergences, as well as permutational MANOVA (PERMANOVA).

Per cent coral mortality per treatment and coral tissue loss were analysed using similar mixed models to algal cover. For growth measures, corals were nested within ambient or enriched plots, but we did not incorporate season as we only analysed change in tissue for corals at the end of the experiment. We calculated tissue loss statistics either excluding or including corals that suffered total colony mortality. Corals that died suffered total colony morality and therefore 100% loss of live tissue area. Including these corals in coral growth analyses resulted in non-normal distributions that could not be corrected via transformations. Therefore, we analysed coral growth both excluding the corals that died, which satisfied normality requirements for the analyses, and including the corals that died. Both analyses produced relatively consistent results (Supplementary Data 5), with the exception that only the interaction of herbivory × nutrient loading was significant in *Porites* corals (rather than each factor also being individually significant) when total colony mortality was excluded. We used a *χ*^2^-test to determine if coral mortality was higher or lower than expected across different seasons given the null hypothesis that coral mortality would be distributed evenly across seasons (25% of total mortality per season).

To assess the impact of algal competition and parrotfish predation on coral mortality for *Porites* spp., (the corals with the highest mortality rates), we used Fisher's exact test to test for differences in the proportion alive versus dead corals across enrichment and/or herbivore exclusion treatments. We also used Fisher's exact tests to determine how factors such as competition with different types of algae (that is, corals with or without algal contact) led to loss/gain of coral tissue or coral death/survival.

Mixed effects models were run using the ‘nlme' package and *post hoc* comparisons conducted using the ‘multcomp' package in R v3.0.0. Fisher's exact tests on contingency tables were run using JMP software (SAS). PCoA of Bray–Curtis divergences and PERMANOVA of benthic community data were conducted in QIIME 1.8.

### Field sampling of coral mucus for microbial analysis

Coral mucus microbial communities were studied in depth for three coral genera common to the study region and most abundant within the plots: *Siderastrea* (*Siderastrea siderea* only), *Porites* and *Agaricia*. We used 16S rRNA gene surveys to study the microbial changes in the coral mucus across the course of treatment, focusing especially on whether detectable microbial changes accompanied specific routes to mortality or tissue loss in corals. (For metadata and sequencing statistics, see Supplementary Data 2; for OTU table in BIOM format, see Supplementary Data 7). We focused on mucus communities because these are thought to provide a barrier against invasion by opportunistic pathogens, and can be sampled non-destructively from the same individuals over time. We deemed other methods, such as collecting live coral tissue, too harmful and invasive to the coral for our goal of monitoring the coral microbiome over the long term.

Coral-associated bacteria and archaea were collected using sterile syringe removal of the coral surface mucus layer on SCUBA. A sterile syringe was used to first agitate the coral and then remove 10 ml of mucus using negative pressure. Once on the surface, the sample was placed in a sterile 15 ml conical tube, frozen on dry ice for the return trip and kept frozen until processing. At a number of time points, 15 ml water sample controls were collected from >1 m above the reef and treated identically to mucus samples in downstream processing. In this study, we did not attempt to evaluate changes in *Symbiodinium* abundance or taxonomy, as it has not yet been established that mucosal abundances of *Symbiodinium* types reflect abundances within tissues, and destructive tissue sampling would preclude time-series analysis of the same coral colonies.

### Quantification of sub-bleaching thermal stress

We calculated cumulative thermal stress using the DHW metric of National oceanic and atmospheric administration (NOAA's) Coral Reef Watch^47^. These calculations rely on a climatological baseline for the mean temperature of the warmest month known as the MMM. We calculated the MMM for our site using the NOAA Pathfinder v5.2 data set, the US official climate data record for sea-surface temperature^45^, and found that the MMM for our site was 29.26 °C (Supplementary Data 6). Data used spanned 1982–2008, and excluded study dates. Temperatures that exceed the MMM by +1 °C are generally regarded as constituting thermal stress^47^. We calculated an MMM +1 °C threshold of 30.26 °C for our site. Temperatures above the 30.26 °C threshold for accumulation of DHWs at our site only occurred during the warm seasons (3 months centred on the warmest month, August).

DHWs represent the extent to which temperatures exceeded the MMM +1 °C in a given season. Temperatures were above 30.26 °C for 7 weeks (total) during all sampling years, resulting in the accumulation of <1 DHW during each of the study years (Supplementary Fig. 10a). In predictions of coral bleaching, accumulation of four DHWs is often associated with minor to moderate bleaching^5^. We saw little bleaching within experimental plots, consistent with sub-bleaching levels of thermal stress. Sub-bleaching thermal stress may nonetheless negatively affect reef health if it increases the abundance and/or virulence of bacterial pathogens. For example, one recent model^15^ proposes that bacterial pathogens may become problematic for corals at temperatures, exceeding the MMM (a lower threshold than the MMM + 1 °C threshold used in coral bleaching studies). We therefore related our microbiological data to both the MMM +1 °C for coral thermal stress during coral bleaching, and the MMM threshold proposed for bacterial pathogens (Supplementary Fig. 10b).

### Coral mucus microbiome sample processing and sequencing

In the laboratory, coral mucus samples were thawed, centrifuged and supernatant decanted. DNA was purified using an organic extraction as previously described^26^. After DNA extraction, microbial 16S amplicon libraries were generated using the primers 515F and 806R, both with added 454 sequencing adaptors and with Golay barcodes added to the reverse primer. Triplicate 25 μl reactions were conducted using the GoTaq Flexi system from Promega (Madison, WI, USA) with the following conditions per reaction: 1 × clear buffer, 1 mM dNTPs, 5 mM MgCl, 1 μM of each primer, 1u Taq polymerase and 1 μl of extracted DNA template. Thermocycling was conducted as follows: 1 cycle of 94 °C for 3 min; 35 cycles of 94 °C for 45 s, 50 °C for 60 s and 72 °C for 90 s; and 1 cycle of 72 °C for 10 min. Amplification success was checked on a 1.5% agarose gel, and successful triplicate reactions were pooled and cleaned using AMPure magnetic beads from Agencourt. Before sequencing, libraries were quantified using a Qubit dsDNA HS kit from Invitrogen and then pooled into equimolar ratios. The pooled library was checked for amplicon length and purity on an Agilent Bioanalyzer 2100 and then sequenced on a 454 Roche pyrosequencer (GSJunior platform) at the Oregon State University's Center for Genome Research and Biocomputing Core Laboratories.

### Microbial community data quality control

The QIIME (v.1.8) software pipeline^65^ was used for quality control, selection of operational taxonomic units and analyses of community diversity (Supplementary Data 2). Sequence libraries were demultiplexed, and sequences with quality scores less than a mean of 35 were removed. Error-correcting barcodes were used to detect and recover sequences whose barcode sequence had exactly one sequencing error. Barcode sequences with two or more errors were removed. Sequences were clustered into operational taxonomic units (OTUs), at a 97% 16S rRNA gene identity threshold using USEARCH 6.1.544, and the subsampled open-reference OTU-picking protocol in QIIME v.1.8 (ref. 65, using greengenes 13_8 as the reference^66^. This OTU-picking protocol clusters all reads, but assigns reference ids to OTUs in greengenes, which can be useful in comparisons across studies. Chimeric sequences were removed with UCHIME. OTUs represented in the overall analysis by only a single count (singletons) account for a large proportion of noisy reads. Because our emphasis was overall community trends (rather than exploration of the rare biosphere of corals), singleton sequences were removed. Representative sequences for each OTU were classified taxonomically according to the greengenes taxonomy version 13_8^66^ using the Ribosomal Database Project (RDP) Classifier software v. 2.2. Sequence alignment and phylogenetic inference for the representative sequence of these OTU is described below in the context of β-diversity analysis.

We took additional steps to account for aspects of the data set unique to host-associated samples. Because coral mucus can contain some amounts of sloughed tissue, we tested whether coral mitochondria were present in any mucus samples. Similarly, because *Symbiodinium* and other photosynthetic microbial eukaryotes frequently inhabit coral mucus, chloroplast sequences are frequently observed in microbial diversity surveys of corals. As our interest was primarily in cellular bacteria and archaea rather than organelles, coral mitochondrial sequences and 16S sequences classified as chloroplasts with at least 70% confidence by the RDP classifier were removed before analysis.

Because we observed that many mitochondrial sequences were not efficiently identified by RDP, we also removed sequences with very high (1−e^−50^) sequence similarity and at least 90% sequence similarity to a reference coral mitochondrial sequence. A table of the coral mitochondrial sequences used is available as Supplementary Data 2 sheet b. These thresholds were selected with care to avoid indiscriminate removal of α-proteobacteria sharing evolutionary ancestry and 16S rRNA gene sequence similarity with mitochondria. The *e*-value for mitochondrial removal was selected by testing several e-values (1−e^−10^,1−e^−30^,1−e^−50^ and 1−e^−100^). For each *e*-value the best BLAST (Basic Local Alignment Search Tool) similarity against NCBI's nr database was examined. We selected the highest (most lenient) *e*-value that removed mitochondria, but not related bacteria. At a 1−e^−50^ BLAST *e*-value threshold, the best BLAST hit of all removed 16S rRNA sequences was coral mitochondria, and this threshold was therefore selected for the screen. The final OTU table after quality control (QC) used for our analysis described in this manuscript can be found in Supplementary Data 7.

### Analysis of microbial β-diversity

Microbial community β-diversity was calculated based on the weighted UniFrac distance matrix^67^. This is a phylogenetic measure of community similarity that takes into account organismal abundance and phylogeny^67^. Phylogenetic trees used to calculate this metric were constructed in QIIME 1.8 (ref. 65) through alignment of representative sequences of each OTU with PyNAST against the greengenes core set alignment^66^, and approximate maximum likelihood phylogenetic inference with FastTree. We considered the pool of distances between samples within each metadata category of interest (for example, algal competition or categories of temperature) using QIIME's make_distance_boxplots.py. Significance was assessed by non-parametric *t*-tests, each with 1,000 Monte Carlo permutations (permutation is important in this instance to account for the non-independence of distances). The effect of this procedure is to ask whether different factors increase the dispersion of communities. PCoA plots of β-diversity were visualized in the Emperor software. When multiple categories (for example, different algal types) were tested for effects on β-diversity, the false discovery rate (FDR) for multiple comparisons was controlled at a threshold of *q*=0.05 using the Bejamini–Hochberg method.

### Comparison of microbial and environmental distance matrices

Mantel tests test for correlation between two distance matrices. For example, a matrix of geographic distances for sample sites might be tested for correlation against a matrix of genetic distances. The partial Mantel test is an extension that tests for correlation between two distance matrices after accounting for the effects of a third, confounding, distance matrix. We used permutational Mantel tests to test whether between-sample variation in continuous environmental factors such as thermal stress, temperature or algal cover correlated with differences in the weighted UniFrac distance matrix^67^ between coral microbial communities. When data on hypothesized confounding factor was available, we used partial Mantel tests to test significance after accounting for the confounding parameter. For example, seasonal variation in algal cover might potentially confound the effects of temperature on microbial communities- partial Mantel tests were used to test for such effects. All Mantel and partial Mantel tests were performed in QIIME 1.8 (ref. 65) using the script compare_distance_matrices.py

### Analysis of microbial community richness and evenness

Microbial community richness, evenness and β-diversity were calculated in QIIME v.1.8. Richness and evenness were calculated using the chao1 and equitability statistics, respectively, in QIIME's alpha_diversity.py, collate_alpha.py and compare_alpha.ph scripts. In each case, the data were repeatedly (10 ×) rarified to 500 reads per sample. Values for chao1 and equitability were calculated for each rarified table, and averaged into a single value and compared across categories. Significance of results was assessed by FDR-corrected non-parametric *t*-tests with 1000 Monte Carlo permutations, using *q*<0.05 as the significance threshold.

Equitability was calculated as defined in the QIIME software package. Specifically, the Shannon entropy (using a base 2 logarithm) was divided by the base 2 logarithm of the number of observed OTUs. Thus, for a sample with *n* OTUs considered in turn (*n*_1_, *n*_2_, *n*_3_, …, *n*_i_), the equitability/evenness was calculated as:


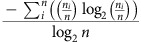


The effects of treatment, temperature, coral genus and individual coral head on richness and evenness were analysed using linear models in R (3.1.1). Seawater temperatures were binned into ‘low temperature', ‘moderate temperature' and ‘high temperature' categories, with thresholds at<24 °C for ‘low temperature' and >30 °C for ‘high temperature'. The high temperature threshold represents the MMM from the NOAA Pathfinder v5.2 SST data set^45^ (see above).

The specific form of these linear models in the R language was: lm(alpha_diversity ∼ treatment * seawater_ temperature_category * coral_genus+individual_coral_head). The significance of each effect was compared with ANOVA (Supplementary Data 3 sheet f).

### Random forest analysis

Random forest analysis is a machine-learning method for supervised classification. We used random forest analysis (Supplementary Data 3 sheets i–j) to (i) determine the conditions under which Synechococcales would be displaced as the most abundant order in the coral microbiome, and (ii) generate a model predicting whether specific coral colonies would lose tissue using ecological (for example, long-term contact with algae from photo series) and microbiological data (the abundance of different bacterial orders).

For both analyses, random forest was conducted in QIIME v.1.8 with the script supervised_learning.py. In both models, the random forest was constructed with 1,000 decision trees and validated by 10-fold cross-validation. This analysis resulted in an overall prediction accuracy, a per-category prediction accuracy and a ranking of feature importance, defined as the amount of accuracy gained or lost when a specific feature was not included in the model. It is worth noting that we employed random forest analysis primarily to provide a summary of the overall predictive power of the data; the internal complexity of the algorithm does not allow inferences to be made about the relationships between variables, beyond their empirical influence on accuracy. For mechanistic insight we rely on other analyses presented here.

In the first model, we sought to predict the dominant (most abundant) order of bacteria in samples given only non-microbial data about the coral colony from which the sample was taken at that point in time. A total of 127 features were used in the prediction including: herbivore exclusion, nutrient addition, coral species (species were converted to binary variables), per cent cover of different macroalgae, turf algae and cyanobacterial mats from quadrat surveys, singly and grouped by functional type (for example, total macroalgae), plot and subplot number, measures of instantaneous and weekly average temperatures and salinities, season (quarter), and the simultaneous presence of parrotfish bites and nutrient enrichment. Given these features, we asked the model to predict the dominant order of microorganism in each DNA sample, with our main purpose being to determine whether the conditions under which *Synechococcus* was dominant were predictable. No microbiological data were included in the input feature table, so all predictions about the microbiology were based on externally observable features.

In the second model, we sought to test whether data about the competitive environment and microbial community composition of specific coral colonies could predict whether they would lose tissue over the 3-year study period. This model included features representing herbivore exclusion, nutrient pollution and coral genus; long-term competition with any algae or (as separate features) with turf algae, visible Cyanobacteria mats*, Dictyota*, *Sargassum* or *Halimeda*; the presence of bleaching, DSS or signs of parrotfish predation; the initial diameter of the coral colony in centimeters; and microbiological data (averaged across all samples from each individual) on the abundance of the phyla Proteobacteria, Cyanobacteria, Bacteroidetes, Firmicutes, Acidobacteria and Actionobacteria overall, and six specific microbial orders of interest due to their prominence in this data set and/or the literature^13^,^41^,^44^ (Synechococchales, Vibrionales, Rhodobacterales, Alteromonadales, Rickettsiales, Rhodospirallales and Pseudomonadales).

### Predicting coral microbiome function

To predict potential functional consequences of observed changes in microbial taxonomy across treatments, functional profiles for each microbial sample were predicted using the PICRUSt tool^40^. This tool uses hidden state prediction, a form of evolutionary modelling closely related to ancestral state reconstruction, to put bounds on genomic copy numbers of each gene family in uncultivated environmental microorganisms, using their position in a reference bacterial phylogeny relative to all bacteria or archaea with sequenced genomes^40^,^68^. These per genome estimates are then combined, taking into account 16S rRNA copy number variation, to estimate a functional profile (or ‘virtual metagenome') for a microbial community based on the information available in all sequenced genomes. The resulting metagenomes have been used in recent analyses of human and environmental microbiomes, and have in at least one study been shown to correlate with overall metabolite profiles (reviewed in ref. 68). The accuracy of the method depends on several factors^40^,^68^, but the availability of reasonably related reference genomes is generally the most important. Availability of such genomes can be summarized through a Nearest Sequenced Taxon Index (NSTI) score. This score is the average branch length between each member of the community (OTU) and its closest sequenced relative, weighted by abundance. In these data, NSTI scores ranged from 0.03 to 0.18, with a mean of 0.11. This was within the range of soil and mammal microbiomes that have previously been predicted with reasonable accuracy (as assessed using paired 16S rRNA/shotgun metagenomes from the same samples)^40^.

We used PICRUSt to predict functional profiles for all 16S rRNA samples, and summarized these profiles of predicted gene family abundance into Kyoto Encyclopedia of Genes and Genomes (KEGG) pathways and functional categories. We then tested whether functional changes in coral microbiomes correlated with either temperature increase, extremes of temperature or the abundance of upright algal cover within a subplot (Supplementary Fig. 5a,b). To test the effects of extremes of temperature (either hot or cold) on coral microbiomes, the imputed abundance of KEGG functional categories (level 3) across samples was regressed against the squared deviation of temperature from 28 °C, which is the average temperature across samples, and also approximates the annual average temperature at the study site over 32 years (calculated from NOAA Pathfinder v5.2^45^). The effects of upright algal cover or increasing temperature were tested by Spearman regression against KEGG functional category abundance. The FDR across KEGG categories was controlled in all cases at *q*<0.05.

### Comparing PICRUSt results with previous experiments

To compare whether the functional categories predicted to change in this field study were broadly consistent with previous laboratory experiments, we compared PICRUSt data with a previous study that exposed corals to several stressors in aquaria and sequenced their metagenomes. KEGG pathways correlated with extremes of temperature (squared deviation from 28 °C, see ‘Predicting coral microbiome function', above) in this study were compared with KEGG categories that increased or decreased in a previous experiment, in which Thurber *et al*.^24^, exposed the Pacific coral *Porites compressa* to thermal stress. Metagenomes from that study^24^ were annotated with KEGG pathways in MG-RAST using default parameters (BLAST *e*-value <10^−5^, 60% coverage and 15% alignable). We compared the 25 categories that increased by >1% with temperature stress to the present study.

### Macroalgal contact as a driver of microbial β-diversity

We sought to test whether the increase in β-diversity with macroalgal contact that we saw in this data set is a common pattern in coral–algal competition. We reanalysed data from a previously published experiment^19^ that studied the effects of macroalgal contact on coral microbiomes of *P. astreoides* (Supplementary Fig. 7c). In that study, macroalgae were placed in direct contact with *P. astreoides* corals, and the microbial communities of the macroalgae, corals without algal competitors and corals with algal competitors assessed using terminal restriction fragment length polymorphism^19^. We translated the table of terminal restriction fragments from this study into a QIIME-compatible .biom format OTU table, and tested whether β-diversity (measured by Bray–Curtis divergence in terminal restriction fragment profiles) was greater in corals exposed to macroalgae than control corals or control algae using analyses described above (see Analysis of Microbial β-diversity).

### Data availability

Processed microbial data are available as Supplementary Data 7 (OTU tables in BIOM format). Metadata for coral microbiome samples are available as Supplementary Data 2 sheets c,d, respectively. Short-read amplicon sequence data (as fasta and qual files) are deposited in the QIITA database, as study id: 10482. Other relevant data are available from the authors.

## Additional information

**How to cite this article:** Zaneveld, J.R. *et al*. Overfishing and nutrient pollution interact with temperature to disrupt coral reefs down to microbial scales. *Nat. Commun.* 7:11833 doi: 10.1038/ncomms11833 (2016).

## Supplementary Material

Supplementary Information Supplementary Figures 1-10, Supplementary Table 1, Supplementary Notes, Supplementary References

Supplementary Data 1 Algal community composition data

Supplementary Data 2 Sequencing statistics

Supplementary Data 3 Microbial orders and their distributions data

Supplementary Data 4 Predicted microbial function data

Supplementary Data 5 Coral mortality and tissue loss

Supplementary Data 6 Temperature and thermal stress data

Supplementary Data 7 OTU Biom file.
