# Supplementary material for: Overfishing and nutrient pollution interact with temperature to disrupt coral reefs down to microbial scales
Source: Nat Commun. 2016 Jun 7;7:11833. doi: 10.1038/ncomms11833 (PMC4899628; doi:10.1038/ncomms11833)
Supplement: Supplementary Information — Supplementary Figures 1-10, Supplementary Table 1, Supplementary Notes, Supplementary References [file ncomms11833-s1.pdf]

Supplementary Information

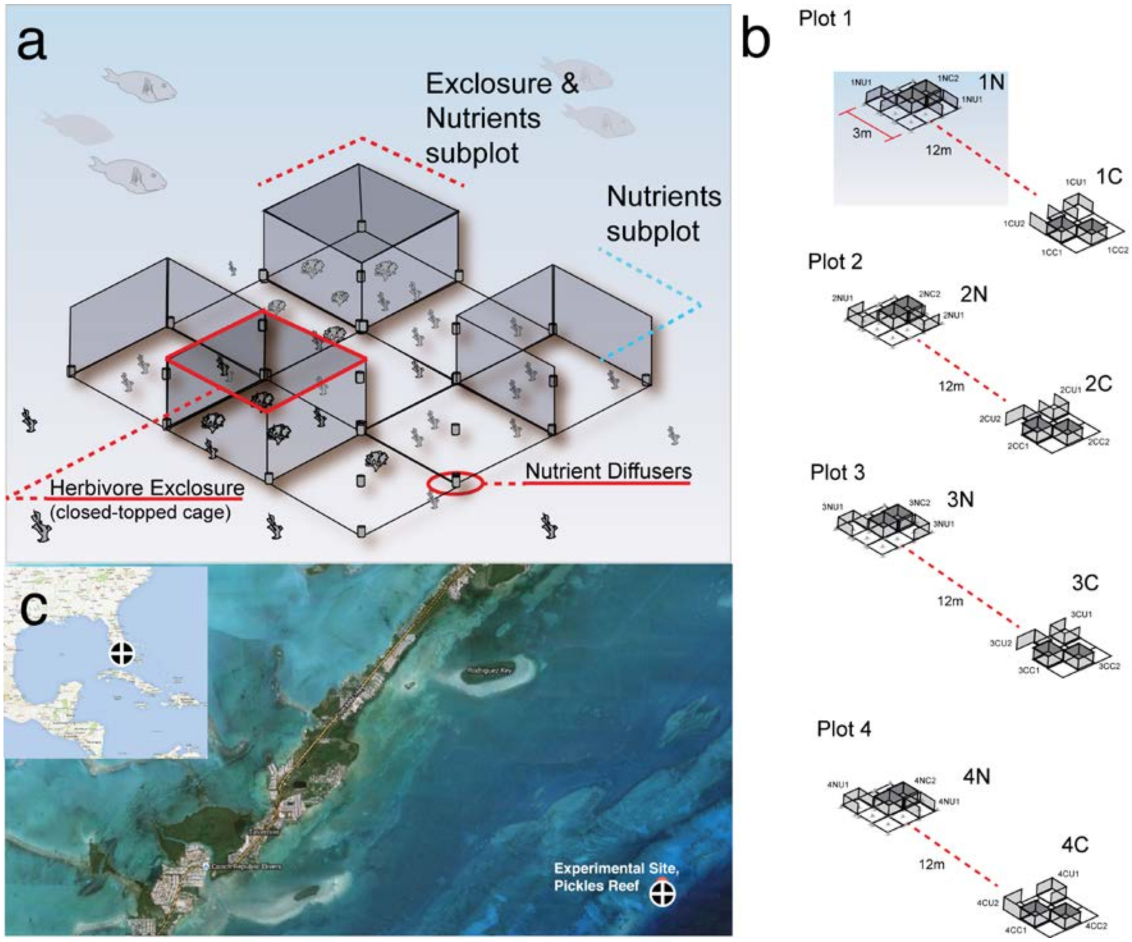

Supplementary Figure 1. Experimental setup.

In order to simulate the effects of overfishing and/or nutrient pollution, 3 m x 3 m (9 m^2) experimental plots were created to exclude herbivores nested within control or nutrient-enriched areas. **a**, Arrangement of each plot. Each 9 m^2 plot was delineated into 1 m^2 plots with metal nails driven into the reef at the corners and center of each plot. Within each 9 m^2 plot, we nested two 1 m^2 herbivore exclosures and two 1 m^2 exclosure controls (three-sided, open-topped partial exclosures, to control for experimental artifacts). All exclosures were made of plastic-coated wire mesh with 2.5 cm diameter holes. This mesh diameter excludes all herbivorous fishes >10 cm total length, but allows in smaller juvenile herbivorous fishes. Exclosures and exclosure controls were constructed within each 9 m^2 plot so as to maximize similarity in initial algal abundance, community structure, and rugosity. For the enrichment treatment, Osmocote® (19-6-12, N-P-K) slow-release garden fertilizer was placed in 15 cm diameter PVC tubes with 10, 1.5 cm holes. These enrichment tubes were attached to each metal nail within the 9 m^2 enrichment plots for a total of 25 enrichment tubes per enrichment plot. Nutrient diffusers were replaced every 30-40 days to ensure continued delivery of N and P. **b**, Arrangement of plots in experiment. Eight of these 9 m^2 plots were constructed, four enriched with nitrogen and phosphorous (N) and four left at ambient levels of nutrient loading to act as control plots

(C). These plots were developed and maintained for ~3 years near Pickles Reef (N 24.99430, W 080.40650). The plot enlarged in panel **a** is shaded in blue in panel **b** in order to show its relationship to the rest of the experiment. Plots are not drawn to scale. **c**, Map of the experimental site. Pickles Reef is a 5-6 m deep spur and groove reef system located just east of Key Largo, Florida, USA, and is representative of coral depauperate habitats common in South Florida. Map data are from Google Maps 2012-2014 and partner INEGI.

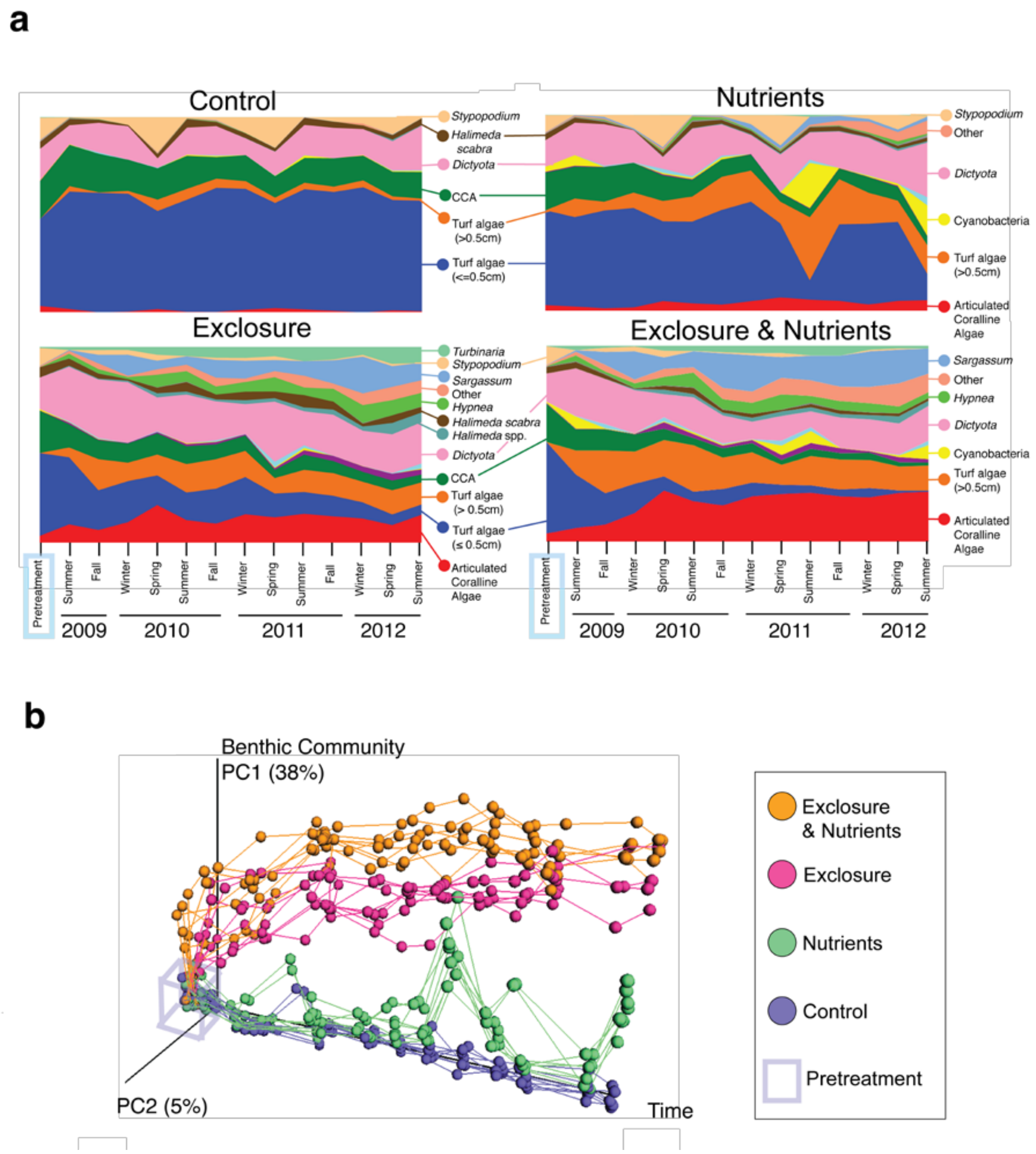

Supplementary Figure 2. Treatments alter algal community composition over time. **a,** Algal abundance over time. Relative cover of each algal taxon or functional group surveyed over time (see also Supplementary Data 1a,b). Cover is normalized to 100% for visual clarity (see Figure 1a for overall changes in absolute cover). CCA, crustose coralline algae. Most algal types responded significantly to exclosure of herbivores or nutrient loading (see Supplementary Data 1c for mixed-effects model results). **b,** Algal community change. Change in algal communities over time is summarized as a PCoA plot of Bray-Curtis divergences between all algal communities exposed to each treatment (the first two PCoA axes are plotted against time). Treatment significantly altered overall community composition (PERMANOVA, 1000 iterations, pseudo-F = 97.11, p = 0.001). Colors represent treatments; the wireframe box surrounds pretreatment communities.

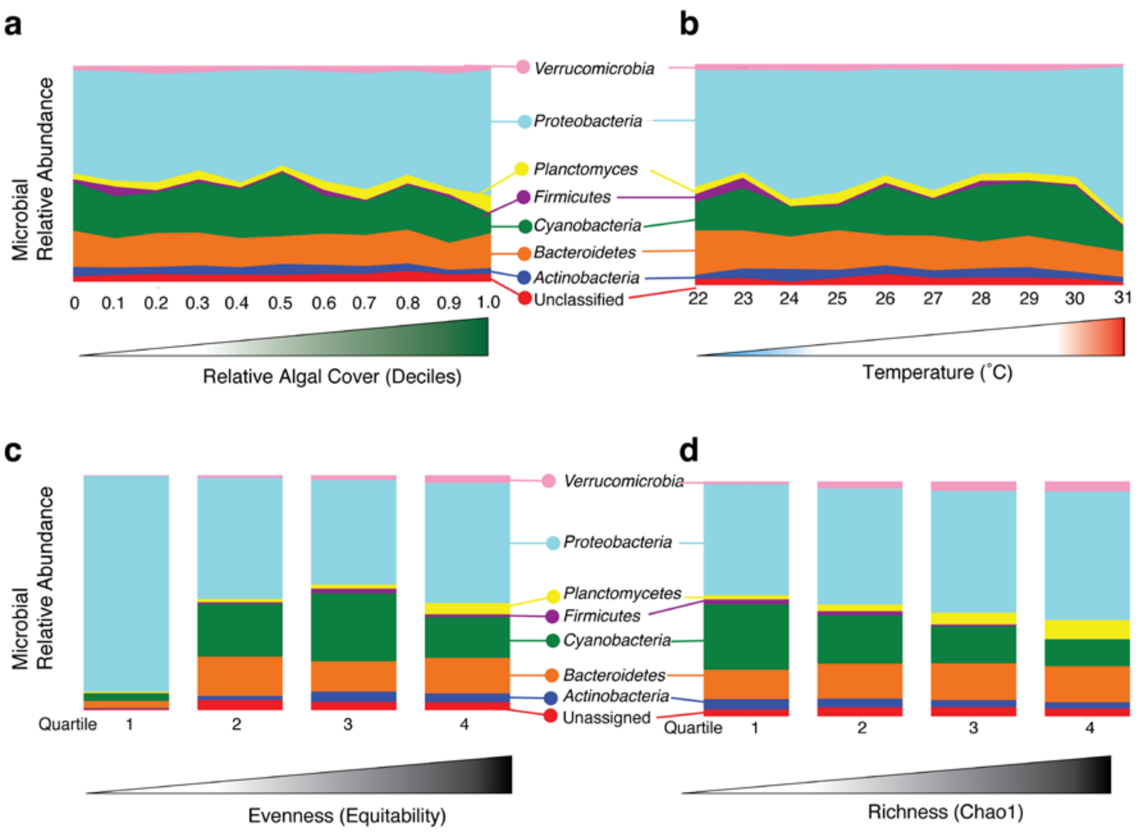

Supplementary Figure 3. Microbial phyla in the coral surface microbiome are altered by algal competition, temperature extremes, microbial community evenness, and microbial community richness.

a-d, Abundance of microbial phyla. Plots display microbial relative abundance in the coral surface mucus layer as a function of deciles of upright algal cover (macroalgae, turf algae, and cyanobacteria), temperature, quartiles of microbial community richness, or quartiles of microbial community evenness. Minor phyla (<1% average relative abundance) are not shown, and only samples with associated temperature or algal metadata are included (n = 435 samples). Significance was assessed by Spearman correlation and a permutational significance test, and multiple comparisons addressed with an FDR q-value threshold of 0.05 (Supplementary Data 3b). **a**, Increasing levels of upright algal cover significantly reduced the abundance of *Actinobacteria*, *Cyanobacteria*, *Bacteroidetes*. In contrast, *Planctomycetes* and Unclassified bacteria increased in abundance as algal cover increased. Elevated temperatures significantly reduced the abundance of *Actinobacteria*, Unassigned microbes, *Bacteroidetes*, and *Firmicutes* (Supplementary Table 3c). **b**, In some cases, taxa appeared to respond to temperature extremes rather than temperature *per se*. This was quantified by regressing microbial abundance against the squared deviation of temperature from 28 °C (Supplementary Table 3d). *Proteobacteria* significantly increased at temperature extremes by this measure, while *Cyanobacteria* decreased. For panels **c** and **d**, all phyla with >1% average abundance were tested for differences in abundance in microbial communities with varying evenness and richness. All phyla tested significantly differed in abundance across quartiles of evenness and richness (Kruskal-Wallis test, $p < 0.05$,

FDR q < 0.05). *Proteobacteria* accounted for 91.9% of the least even communities (1st quartile of evenness). In contrast *Proteobacteria* made up only 44-52% of communities in the 2nd, 3rd, and 4th quartiles of evenness, where *Cyanobacteria* were more abundant. More taxonomically rich communities had significantly fewer *Cyanobacteria* (the richest quartile was 10.7% *Cyanobacteria*; the least rich 24.0%) and *Actinobacteria* (2.7% in the most rich quartile *vs.* 4.0% in the least rich quartile) but more *Proteobacteria* (52.7% vs. 46.4%), *Bacteroidetes* (14.7% vs. 13.1%) and *Planctomyces (*7.6% vs. 1.8%).

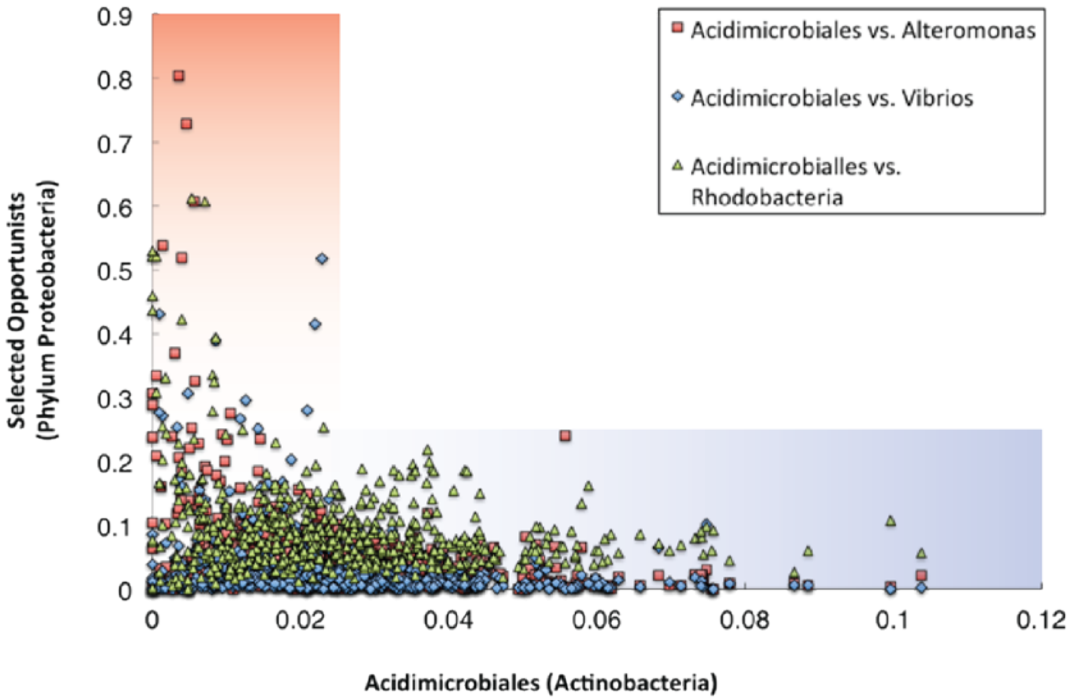

Supplementary Figure 4. *Actinobacteria* and blooms of opportunists in the coral mucus microbiome.

Each point represents a single 16S rRNA sample from coral mucus. Coordinates plot the abundance of *Acidimicrobiales* (Actinobacteria) on the x-axis vs. the relative abundance of *Alteromonadales, Vibrionales,* or *Rhodobacterales* on the y-axis. Outbreaks of these opportunists to abundances above 25% of the community (horizontal blue gradient) were not observed when *Acidimicrobiales* were present at >2.5% abundance. Reductions of *Acidimicrobiales* below ~2.5% appear to allow blooms of *Vibrionales, Rhodobacterales,* and *Alteromonadales* opportunists that rose as high as 80% of total community composition (vertical red gradient). See Supplementary Data 3e for data on environmental conditions favoring dominance by specific groups.

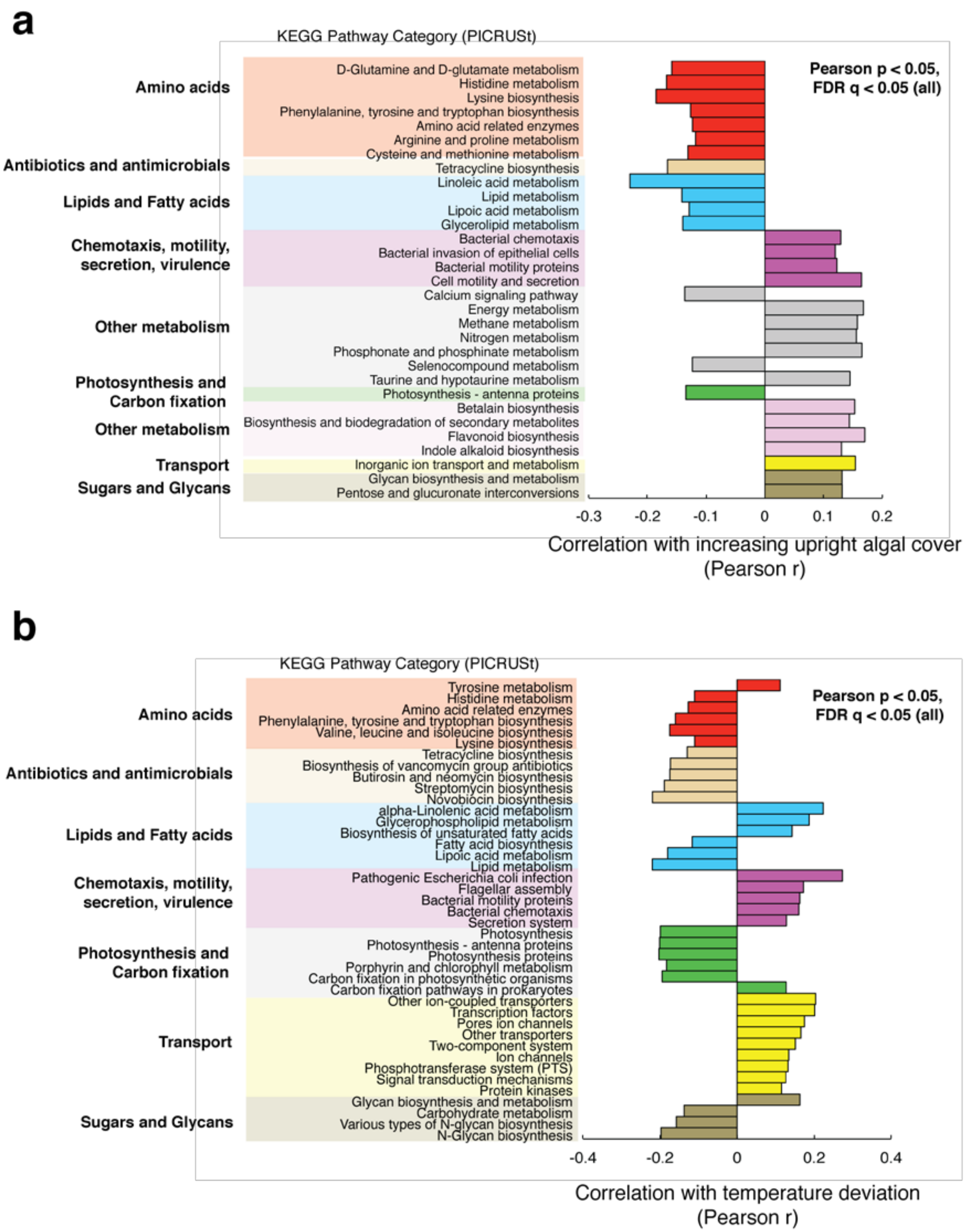

Supplementary Figure 5. PICRUSt predicted changes in coral microbiome gene function correlated with increasing upright algal cover or temperature extremes. **a-b,** Predicted microbial functions. Functional profiles (KEGG orthology groups) for each sample were imputed based on comparison of microbial community composition with sequenced bacteria and archaea in the PICRUSt software package. These profiles were summarized into KEGG categories. Selected KEGG categories that showed

significant positive or negative correlations (Pearson correlation, FDR $q < 0.05$) with increasing upright algal cover (panel **a**) or temperature extremes (panel **b**) are shown. Temperature extremes were calculated as the mean squared deviation from 28 °C , a non-stressful temperature that reflected the mean temperature across samples (this also approximated the overall annual average at the site)**.** For the full set of categories significantly correlated with upright algal cover or temperature extremes, see Supplementary Data 4a,b.

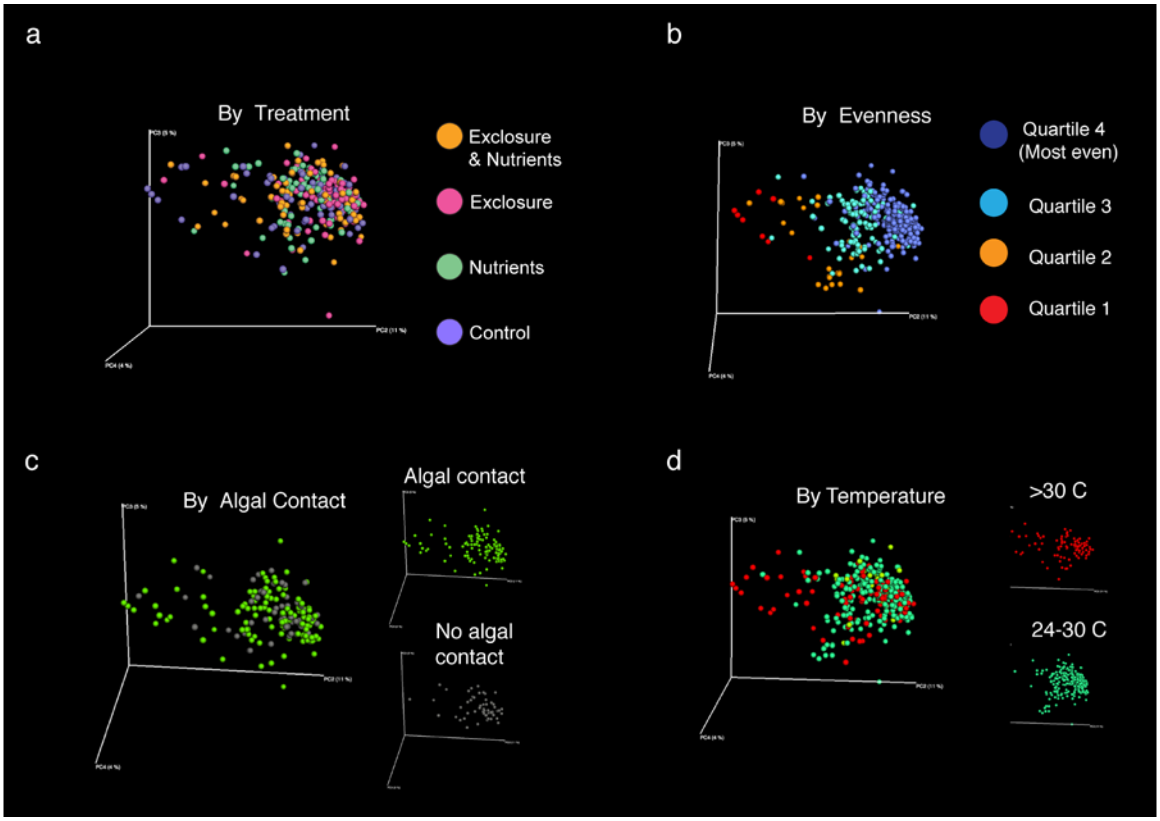

Supplementary Figure 6. β-diversity of coral microbiomes relative to treatment, evenness, algal competition, or thermal stress.

a-d, PCoA plots of coral mucus microbiome β-diversity using Weighted UniFrac distances between 16S rRNA gene amplicon libraries, showing PC axes 2,3 and 4 (PC 1, which captures differences between *Synechococcus* and *Proteobacteria* dominated communities is shown in Fig. 2). The plots are colored by treatment (panel **a**), microbial community evenness (panel **b**) algal contact (panel **c**), or temperature (panel **d**). Points representing samples where a metadata classification was unavailable were excluded from plots, for example in cases where coral-algal contact could not be assessed. Subpanels reproduce main plots, but separated by selected metadata categories. Evenness in panel **b** represents quartiles of microbial community equitability. While no clustering by treatment was observed in panel **a**, microbial β-diversity was significantly higher in corals contacting algae than in those that did not (non-parametric t-test on Weighted Unifrac distances, p = 0.001) in panel **c**, and in panel **d** corals at high temperatures (>30 °C; p = 0.001) or low temperatures (<24 °C; p = 0.002, not shown) had higher microbial β-diversity relative to samples collected when sea surface temperatures were between 24-30 °C. Thus, interactions with algae and temperature extremes increase β-diversity in coral microbiomes.

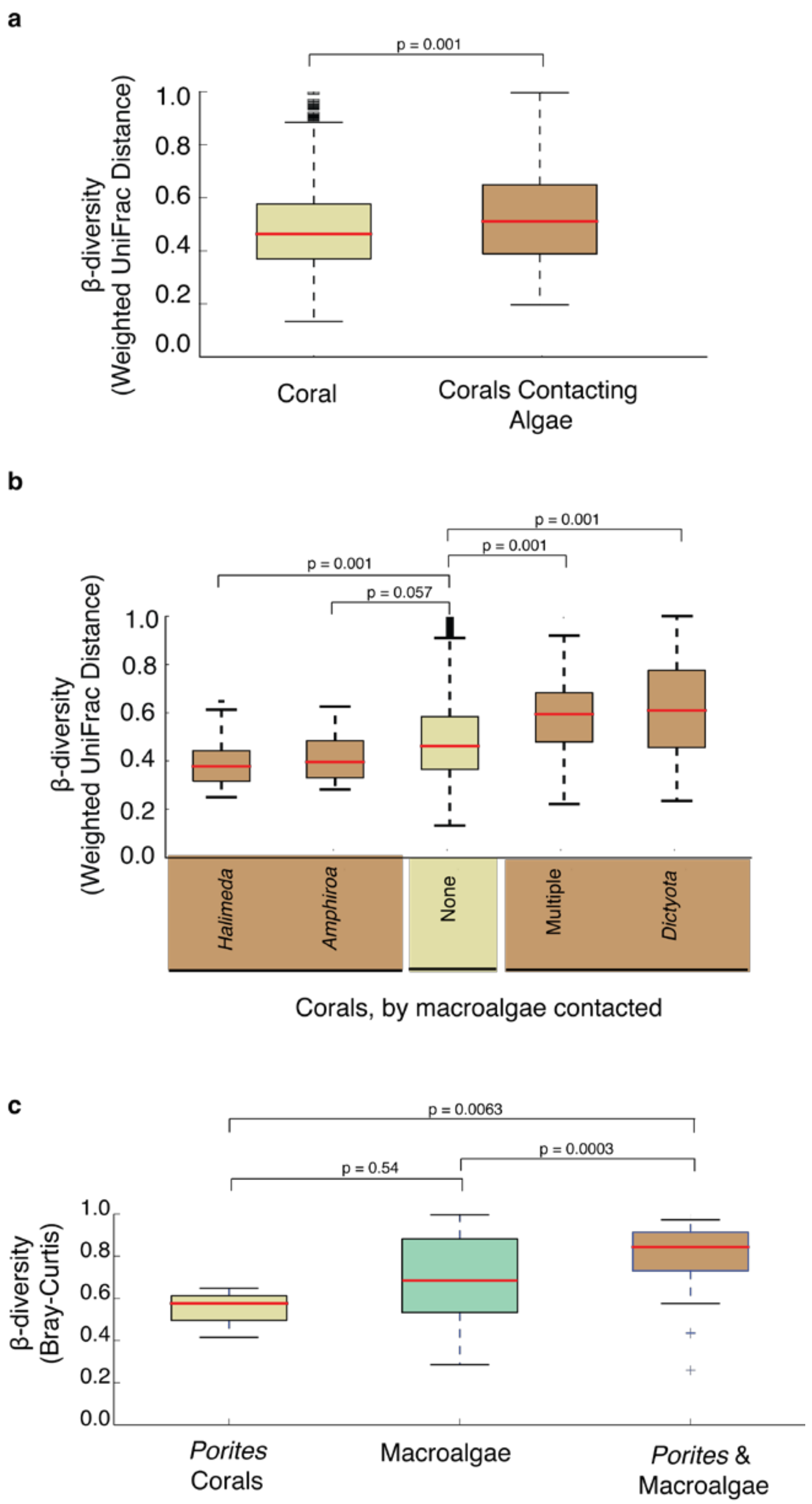

Supplementary Figure 7. Coral microbiome β-diversity as a function of competition with macroalgae.

a-b, Effects of algal competition on coral microbiome β-diversity in this experiment are shown for all macroalgal contacts (panel a) and contact with prevalent macroalgal genera (panel **b**). All microbial distances are Weighted UniFrac distances. Overall, contact with macroalgae increased microbial beta-diversity (non-parametric t-test, 1000 replicates, $p = 0.001$). When split into categories by macroalgal genera, large significant increases in microbial variability were observed in corals contacting *Dictyota* algae (non-parametric t-test, 1000 replicates, $p = 0.001$) or multiple types of macroalgae ($p = 0.001$). In contrast, competition with *Amphiroa* algae marginally reduced β-diversity ($p = 0.057$) while corals in competition with *Halimeda* showed significantly reduced β-diversity ($p = 0.001$). **c,** Data on microbial β-diversity caused by algal contact based on a re-analysis of Vega Thurber *et al.*, 2012[23]. In that experiment, samples were collected from macroalgae, *Porites astreoides* corals alone, or *P. astreoides* placed in direct competition with macroalgae. Box plots show β-diversity of corals, algae, or corals in competition with algae. Algal contact significantly increases coral microbiome β-diversity above that of either coral alone or algae alone. P-values reflect Bonferroni-corrected permutational t-tests of Bray-Curtis distances between samples. Bray-Curtis distances were used here as measures of β-diversity because Weighted UniFrac could not be calculated for T-RFLP data.

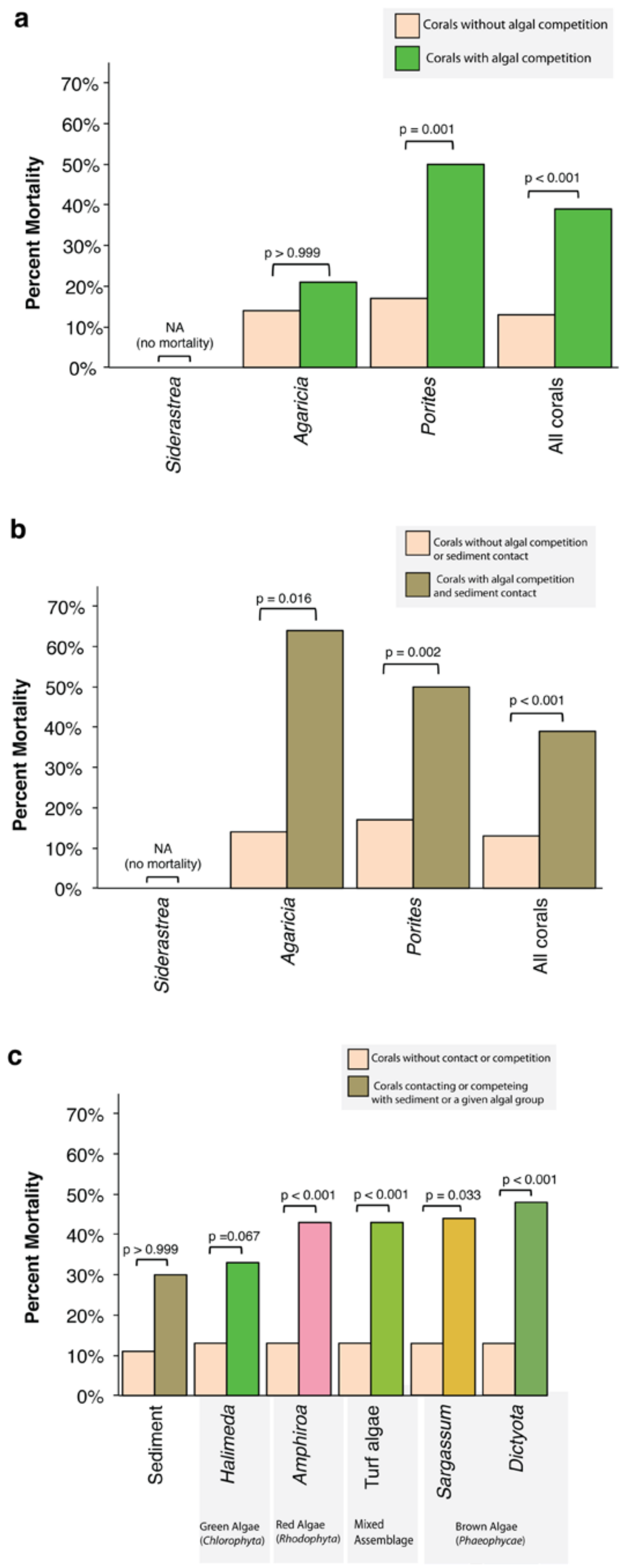
a
Corals without algal competition
Corals with algal competition
Percent Mortality
70%
60%
50%
40%
30%
20%
10%
0%
NA
(no mortality)
p > 0.999
p = 0.001
p < 0.001
Siderastrea
Agaricia
Porites
All corals
b
Corals without algal competition or sediment contact
Corals with algal competition and sediment contact
p = 0.016
p = 0.002
p < 0.001
c
Corals without contact or competition
Corals contacting or competeing with sediment or a given algal group
p > 0.999
p =0.067
p < 0.001
p < 0.001
p = 0.033
p < 0.001
Sediment
Halimeda
Amphiroa
Turf algae
Sargassum
Dictyota
Green Algae (Chlorophyta)
Red Algae (Rhodophyta)
Mixed Assemblage
Brown Algae (Phaeophycae)

144

Supplementary Figure 8. Coral mortality as a function of contact with algae or sediment.

Panels show the percentage of corals that died over the course of the experiment as a function of contact with sediment or different types of algae**. a,** Coral morality by coral genus over the course of the experiment as a function of algal contact. *Porites* corals showed significantly elevated mortality with algal contact, while *Agaricia* corals showed a similar pattern but no significant difference. *Siderastrea* corals suffered no mortality regardless of algal contact. **b,** coral morality by coral genus over the course of the experiment as a function of algal and sediment contact. *Agaricia* corals showed greater susceptibility to the combination of sediment and algae than to algae alone. In most cases, competition with algae or contact with sediment increased coral mortality. **c,** coral mortality subdivided by algae contacted**.** In some cases, corals contacted more than one type of algae or algae and sediment making these categories not mutually exclusive. Many algal taxa did not contact corals frequently enough for meaningful statistics (e.g. cyanobacteria, *Stypopodium zonale*). P-values reflect Fisher's Exact Test.

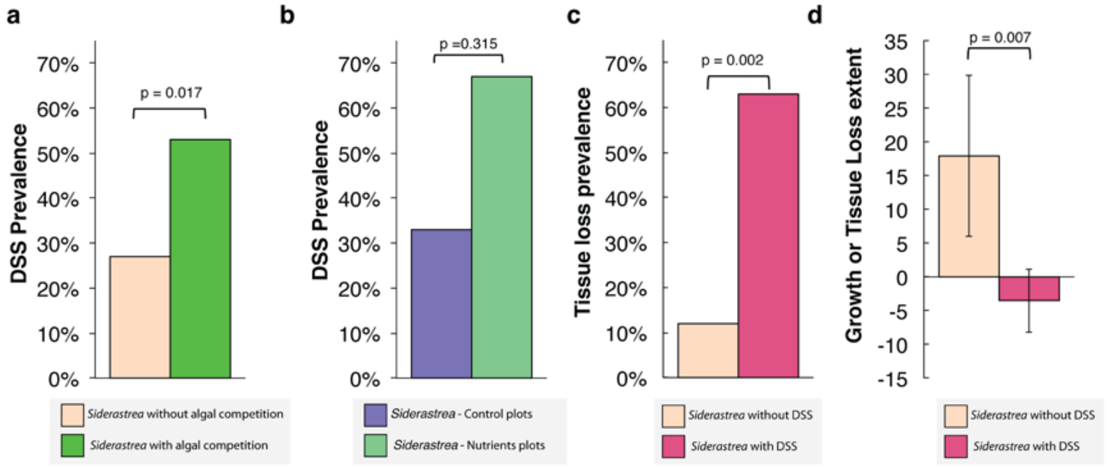

Supplementary Figure 9. Dark Spot Syndrome in *Siderastrea* corals increases with algal contact and is associated with increased prevalence and extent of tissue loss.

a, Prevalence of Dark Spot Syndrome (DSS) across *Siderastrea siderea* corals contacting or not contacting algae. Algal contact increases the prevalence of DSS. **b**, Prevalence of DSS in Control vs. Nutrient Enriched plots. Nutrient enrichment did not significantly increase DSS as we had shown in Vega Thurber *et al*. (2014), although the pattern is the same as in our previous study. These data reflect only corals within the plots themselves. Our previous work showed similar and highly significant increases in the prevalence of DSS in radial surveys around the same enriched plots studied here that included 3-4 times more corals than within these plots. Thus, the lack of significance within plots may reflect the lower number of *Siderastrea* corals within plots than in radial surveys around the plots as in [7]. **c**, Tissue loss prevalence in *Siderastrea* corals with or without DSS. Corals with visible DSS were more likely to lose tissue. **d**, Average extent of growth (positive numbers) or tissue loss (negative numbers) in corals with or without DSS. While *Siderastrea* without DSS gained tissue on average, those with DSS lost tissue. Statistics are from Fisher’s exact test **a-c** or ANOVA **d**.

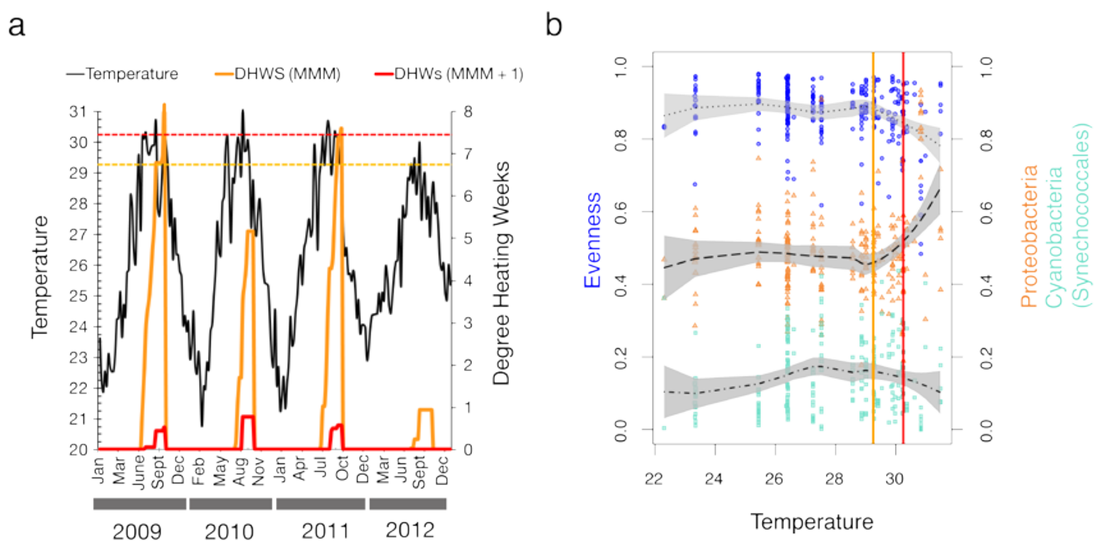

Supplementary Figure 10. Temperature, thermal stress, and effects on the coral microbiome. a, Temperature time-series based on the Pathfinder V5.2 dataset (Methods). The left vertical axis and thin solid black lines shows temperatures in °C. The horizontal orange dotted line indicate the maximum monthly mean (MMM) temperature of 29.26 °C. This is calculated as the average temperature of the warmest month in available climatological data (1982-2008; i.e. excluding the study period). The red horizontal dotted line indicates the MMM +1 °C (30.26 °C), which is often used as a temperature threshold at which coral thermal stress begins to accumulate in predictions of coral bleaching. For purposes of predicting coral bleaching, coral thermal stress is typically measured in units of Degree Heating Weeks (DHWs, °C-weeks). DHWs are usually calculated relative to the MMM +1 °C, and are the accumulation of temperatures above this threshold. As a hypothetical example, if temperatures exceeded the MMM + 1 by 0.2 C for 3 weeks, 0.6 DHWs would accumulate. The accumulation of degree heating weeks due to temperatures above the MMM + 1 °C is shown by the solid red line and the secondary vertical axis on the right hand side of the plot (DHWs (MMM +1)). Because vulnerability to pathogenic bacteria is thought to occur at lower levels of thermal stress than coral bleaching, we also plotted DHWs calculated relative to the MMM. This is shown by the orange line (DHWs (MMM)). We emphasize that all DHW values presented in the main text are calculated based on the MMM + 1 °C (red lines). **b,** Coral microbiomes vs. temperature. Microbial community evenness (left axis, blue circles) and the relative abundance of *Proteobacteria* and *Synechococcus* cyanobacteria (right axis, orange triangles and cyan squares, respectively). The x-axis shows sea-surface temperatures. Regression lines show the loess regression (span = 0.25) for each data series (evenness, dotted lines; *Proteobacteria*, dashed line; *Synechococcus* dot-dashed line). Gray shading around each line indicates twice the standard error of the regression. Vertical lines indicate metrics from the thermal stress calculation. The orange vertical line is the MMM, the red one is the MMM + 1 °C. Notably, the abundance of *Proteobacteria* increase, and overall community evenness decrease around the MMM of 29.26 °C.

Taxonomy (Microbial Order)	Percent Change in Relative Abundance Compared to Control		
	Nutrient	Exclosure	Exclosure & Nutrient
Archaea Crenarchaeota Thaumarchaeota Cenarchaeales	-20.79%	61.83%	0.78%
Acidobacteria Sva0725 Sva0725	4.75%	111.13%	45.47%
Bacteroidetes Bacteroidia Bacteroidales	-19.33%	41.36%	9.40%
Bacteroidetes Flavobacteriia Flavobacteriales	-3.31%	29.07%	22.62%
Chlamydiae Chlamydiia Chlamydiales	49.44%	149.04%	60.60%
Cyanobacteria Synechococcophycideae Synechococcales	5.42%	-22.24%	-21.25%
Fusobacteria Fusobacteriia Fusobacteriales	-19.08%	39.01%	-16.80%
Gemmatimonadetes Gemm-2	31.21%	142.33%	102.66%
Lentisphaeraec Lentisphaeria Lentisphaerales	-16.45%	32.89%	30.42%
Planctomycetes BD7-11	-32.83%	125.51%	-17.17%
Planctomycetes C6od113	-17.83%	107.33%	41.01%
Planctomycetes OM190 agg27	-4.80%	58.00%	21.55%
Planctomycetes Planctomycetia Pirellulales	-10.75%	70.81%	11.03%
Proteobacteria Alphaproteobacteria Rhodospirillales	-17.96%	54.61%	86.92%
Proteobacteria Alphaproteobacteria Rickettsiales	83.04%	14.69%	-5.89%
Proteobacteria Deltaproteobacteria Myxococcales	-6.81%	28.66%	13.02%
Proteobacteria Deltaproteobacteria NB1-j	-3.92%	109.96%	67.54%
Proteobacteria Deltaproteobacteria Spirobacillales	-6.86%	43.69%	22.35%
Proteobacteria Gammaproteobacteria Chromatiales	-32.93%	40.63%	-5.00%
Proteobacteria Gammaproteobacteria HOC36	-5.57%	5.91%	65.07%
Proteobacteria Gammaproteobacteria Marinicellales	-15.52%	23.66%	18.56%
Proteobacteria Gammaproteobacteria Thiohalorhabdales	-45.08%	17.75%	-7.73%
Proteobacteria Gammaproteobacteria Thiotrichales	-15.76%	37.10%	11.54%
Proteobacteria Gammaproteobacteria; Alteromonadales	12.96%	-6.25%	-6.25%
Spirochaetes Spirochaetes Spirochaetales	0.60%	111.06%	18.23%

Positive	Negative
>100%	0 to -15%
50 to 99%	-16 to 30%
0 to 49%	-31 to -50%

Supplementary Table 1. Response of microbial orders to treatment. Table reports microbial orders that were significantly different (Kruskal-Wallis, FDR $q \leq 0.05$) across treatments. Values report percent changes compared to corals in control plots. Cold colors are reductions compared to controls and hot colors represent increases compared to controls. In general, corals in herbivore exclosures had large elevations in many taxa as compared to controls while nutrient pollution caused small to moderate reductions in various taxa. The Taxonomy column gives phylum, class, and order based on Greengenes taxonomy.

Supplementary Notes

Natural history of the study site

This experiment was conducted in the area of Pickles Reef (N 24.99430, W 80.40650), located east of Key Largo, Florida in the United States. The Florida Keys reef tract consists of a large bank reef system located approximately 8 km offshore of the Florida Keys, USA, and paralleling the island chain. Our study reef is a 5-6 m deep spur and groove reef system within this reef tract. The reefs of the Florida Keys have robust herbivorous fish populations[1] and are relatively oligotrophic[2]. Coral cover on most reefs in the Florida Keys, including our site, is 5-10%, while macroalgal cover averages ~15%, but ranges from 0-70% depending on location and season[3-5]. Parrotfishes (*Scaridae*) and surgeonfishes (*Acanthuridae*) are the dominant herbivores on these reefs as fishing for them was banned in 1981. The other important herbivore on Caribbean reefs, the urchin *Diadema antillarum*, remains at low densities across the Florida Keys following the mass mortality event in 1982-3[6].

Tests of the experimental design and implementation

Nutrient enrichment in seawater and algal tissue samples

Sampling of water column nutrients showed that enrichment increased both dissolved inorganic nitrogen (3.91 μM vs. 1.15 μM in enriched vs. control) and soluble reactive phosphorus (0.27 μM vs. 0.035 μM in enriched vs. control) in the water column[7]. Levels of both DIN and SRP in the control plots were within the range of concentrations for offshore reefs, as measured in a 15-year water-monitoring program in the Florida Keys[8]. Levels of DIN and SRP in the enriched treatment were similar to those reported from other anthropogenically-impacted reefs located around the world[9]. Additionally, nitrogen concentrations in the tissues of the common alga *Dictyota menstrualis* were 20% higher in the enriched plots compared to the control plots, suggesting that the nutrients from the enrichment were consistently available to benthic organisms [7] .

Herbivorous fish community assessments

Herbivorous fish biomass averaged 50.9 ± 5.7 g/m^2 over the course of our sampling, which is among the higher values seen across the Caribbean[10,11]. There were no differences in biomass across different sampling periods (ANOVA: $F = 0.23$, $p = 0.880$). On average, parrotfishes and surgeonfishes represented 55% and 45% of total herbivorous fish biomass respectively. We did not quantify *D. antillarum* as they were rarely encountered.

Tests for unintended experimental exclosure effects

One potential concern with using exclosures to manipulate herbivore access is that they can potentially alter water flow regimes or sedimentation inside of the full exclosures, creating experimental artifacts in patterns of algal community structure. We saw no differences between full exclosures, exclosure controls, or open areas for either sedimentation rates measured via sediment traps (e.g. [12]; one-way ANOVA, $F = 0.37$, $p = 0.70$) or for bulk flow rates measured via clod cards (e.g. [13]; one-way ANOVA, $F = 3.01$, $p = 0.10$). Exclosures were scrubbed every 4-6 weeks to minimize growth of fouling organisms and minimize changes in flow. However, these exclosures do decrease light

availability to the benthos by 15% (e.g. [14]). Given that the light availability common at these shallow depths saturates the photosystems of primary producers[15], the slight decrease in light availability likely had minimal impact on primary production or interactions among benthic organisms.

Further, we saw no differences in damselfish densities (primarily the bicolor damselfish *Stegastes partitus*) between exclosures vs. exclosure controls (one-way ANOVA, $F = 0.43$, $p = 0.54$) or between enriched vs. ambient nutrient plots (one-way ANOVA, $F = 0.16$, $p = 0.71$). Thus, these territorial and aggressive fishes that can impact how larger herbivorous fishes feed (e.g. [16]) likely did not differently affect the treatments. Further, we are confident that our treatments imparted minimal caging artifacts as others have shown minimal artifacts from using similar designs (e.g. [17-20]).

Benthic community surveys

Shifts in algal community composition

Increases in total algal cover corresponded to alterations in algal community composition (Supplementary Figure 2a,b). Control plots were marked by high abundances of closely cropped filamentous turf algae and crustose coralline algae. On healthy reefs a high abundance of these two algal functional groups, along with low abundance of macroalgae, makes for productive reefs and prime habitat for coral recruitment, growth, and reproduction[21]. Nutrient pollution and/or herbivore exclusion decreased cover of both of these groups while also increasing the abundance of algae that can be harmful to corals. Herbivore exclusion in general led to increases in several groups of algae, such as *Sargassum* spp., *Amphiroa* spp., tall filamentous turf, *Dictytoa* spp., and *Halimeda* spp., known to both harm corals in direct competition[22,23] and strongly impact the coral microbiome[23-25]. Nutrient enrichment also increased the abundance of important algal competitors such as *Dictyota* spp., tall filamentous turf, and *Lyngbia* spp. cyanobacteria, which are noted for producing chemicals harmful to corals and their microbes[26].

Microbial community analysis

Results of coral microbiome function prediction

When we assessed predicted microbiome function in response to algal abundance, 76 functional categories were significantly correlated with upright algal cover (Pearson correlation, FDR $q < 0.05$), with correlation coefficients ranging from -0.23 to 0.18 (Supplementary Figure 5a, Supplementary Data 4a). High levels of macroalgae increased functional categories associated with opportunism (Bacterial Invasion of Epithelial Cells, Bacterial Motility Proteins, Bacterial Chemotaxis, Cell Motility and Secretion), and decreased the abundance of pathways involved in antibiotic production (here the single category tetracycline biosynthesis). The strongest single correlate of increased algal cover was a predicted decrease in microbial genes for linoleic acid (C18:2, *cis-cis*-9,12) metabolism.

Extremes of temperature significantly altered the predicted abundance of 124 KEGG Pathways (Pearson correlation, $p < 0.05$, FDR $q < 0.05$). Extremes of temperature increased the predicted genomic abundance of pathways for bacterial chemotaxis, motility, environmental sensing and secretion often observed to increase with opportunism (Supplementary Figure 5b, Supplementary Table 4b). Previous work has

shown that elevated temperature increases DMSP release from the coral holobiont, which in turn guides chemotaxis by several marine microorganisms[27], including the coral pathogen *Vibrio corallyticus*, to the coral surface[28].

Conversely, five categories of antibiotic/antimicrobial production decreased in extreme temperature (Novobiocin biosynthesis, Streptomycin biosynthesis, isoquinoline alkaloid biosynthesis, Biosynthesis of vancomycin group antibiotics, Tetracycline biosynthesis), as did several pathways associated with photosynthesis (Photosynthesis proteins, photosynthesis- antennae proteins, carbon fixation in photosynthetic organisms, porphyrin and chlorophyll metabolism). These changes may reflect shifts from a community rich in phototrophs (*Synechococcus*) and defensive symbionts (e.g. *Actinobacteria*) to one more strongly dominated by a variety of mostly proteobacterial opportunists.

Comparison of predicted functional profiles for corals under a range of temperatures with metagenomic data from laboratory temperature manipulations

KEGG categories associated with increasing temperature in the present study tended to change in the same direction as observed in Vega Thurber *et al*., 2009[29]. These commonalities were striking given that one data set represents genome-based predictions from field 16S rRNA data, while the other represents pooled metagenomes from a laboratory experiment. To quantify these trends we used sign tests to compare the direction of change with temperature for the 24 level 3 KEGG functional categories that changed by >1% with elevated temperature in Vega Thurber *et al*., 2009. We compared these categories with either: a) the set of 13 KEGG categories significantly associated with temperature in this study (dropping pathways that did not significantly change here) or b) all 24 KEGG categories enriched by temperature in the previous study.

Regardless of the method used, KEGG categories showed a significant tendency to change in the same direction with temperature. Among the set of all 24 KEGG pathways increased by temperature in Vega Thurber 2009, 17 of 24 changed in the same direction in this study. Thus, the direction of change with temperature was significant overall between these two very different experiments (one-sided sign test, 17 successes, 24 trials, $p = 0.032$). The 13 KEGG Pathways that changed by >1% in Vega Thurber 2009 and also changed significantly in this study (Pearson correlation, FDR $q < 0.05$) also had a significant tendency to change in the same direction (one sided sign test, 10 trials, 13 successes, $p = 0.046$).

Confirmation of macroalgal contact as a driver of microbial beta diversity

To confirm that direct macroalgal contact was the driver of changes in the microbial β-diversity seen in this work, we reanalyzed data from a previous experiment[23] at the same study site that placed replicate *Porites* corals in contact with macroalgae. Three categories from that experiment were compared: corals alone, algae alone, or corals in competition with algae (Supplementary Figure 7c). Corals in contact with macroalgae showed higher β-diversity than corals without macroalgae (Bonferroni-corrected permutational t-test, $p = 0.009$). β-diversity in corals in contact with macroalgae was also greater than for the algae themselves (Bonferroni-corrected permutational t-test, $p = 0.003$).

Effects of algal competition on microbial beta-diversity within coral genera

To test whether increasing β-diversity with algal competition is a consistent feature of the coral genera in our study, the effects of algal competition on microbial β-diversity was assessed within each coral genus. In each case, mean microbial community β-diversity was greater when corals were in competition with macroalgae, although when *Porites* was considered alone this difference was not significant (permutational t-tests; *Agaricia*, p = 0.006; *Siderastrea*, l p = 0.005, *Porites* p = 0.74). However, the variability imparted to *Porites* microbiomes from parrotfish bites and nutrient enrichment could have confounded the impact of macroalgae. Similarly, competition with *Dictyota* algae significantly increased microbial β-diversity in all coral genera (*Agaricia* p = 0.001; *Siderastrea* p = 0.001; *Porites* p = 0.002). These findings do not exclude the likely possibility that the microbes associated with many coral genera have partially species-specific interactions with particular algae.

Comparison of temperature vs. seasonal effects on microbial beta diversity

Temperature was an important correlate of many aspects of microbial community structure. Because temperature was not experimentally manipulated, and many other oceanographic parameters fluctuate seasonally, we sought to test whether apparent changes in temperature might be due to unrelated seasonal changes.

We first tested whether temperature increased microbial β-diversity within seasons as well as between seasons. We reasoned that if overall seasonal changes drove microbial β-diversity, and correlations with temperature were an incidental byproduct, then within-season effects of temperature on microbial β-diversity should be weak or non-existent. Instead, we found that even within summer and fall samples (considered separately), high temperatures (>30 °C) resulted in greater β-diversity than non-stressful temperatures (24-29 °C), indicating that thermal stress influences microbial communities within as well as between seasons (Summer: p = 0.001, permutational t-test; Fall p= 0.001, permutational t-test). Consistent with either warm or cold temperatures disrupting coral microbiomes, low (< 24 °C) temperatures in winter were associated with significantly greater β-diversity than 24-29 °C winter samples (p = 0.003, permutational t-test).

To address the possibility that some other seasonal environmental factor besides short-term temperature changes might be the main driver of microbial β-diversity, we examined 40 environmental parameters measured seasonally by the SERC Water Quality Monitoring Network in South Florida, including dissolved inorganic nitrogen, total organic carbon, chlorophyll a, turbidity, SiO_2, etc. (Supplemental Data 3k). To test whether differences in these parameters might explain microbial β-diversity, we constructed Euclidean distance matrices for each parameter across samples, and used Mantel tests, a permutational procedure for comparing two distance matrices, to test whether any of these environmental parameters significantly correlated with microbial β-diversity. No correction for multiple comparisons was performed in this instance, since we did not wish to miss a relevant parameter that might falsify our interpretation of the role of temperature.

Among the measured parameters and temperature, only three correlated significantly with microbial β-diversity. Daily temperature had the greatest influence (r = 0.127, p = 0.01), with surface measurements of total organic carbon (TOC; r = 0.045, p = 0.02), and turbidity (r = 0.09, p = 0.04) playing secondary roles. Notably, although daily

temperature measurements (HCOM_temp_0m_degrees) were well correlated with microbial community structure, seasonal temperature measurements (collected by SERC quarterly at a single time point per season) were not (SERC_TEMP_B). This may indicate that short-term changes in temperature are important, above and beyond typical seasonal temperature trends.

A final possibility that we considered was that the apparent effects of temperature on β-diversity might be explained by the relatively modest seasonal variation in upright algal cover (which includes both tall turf algae and macroalgae). To disentangle the effects of temperature from upright algal cover on microbial β-diversity, we conducted a partial Mantel test examining the relationship between temperature and microbial β-diversity while normalizing for the effect of upright algal cover. We found that temperature was still significantly correlated with β-diversity (Partial Mantel test; $r = 0.12$ $p = 0.01$) after accounting for the effects of upright algal cover (Supplemental Data 3k).

We do not interpret these results to mean that temperature is the only influence on microbial communities. For example, oceanographic parameters not significant on a quarterly basis may have important short-term or spatially localized effects that could be uncovered with high-resolution sampling. However, these results taken together argue against the possibility that the observed correlations between temperature and microbial community structure are artifacts of seasonal fluctuations in water chemistry or algal cover. Instead, short-term temperature variation appears to be an important factor influencing coral microbiome stability. This analysis also identified seasonal variation in dissolved organic carbon and turbidity as additional influences on coral microbial community structure, consistent with the effects of these parameters on corals and coral microbiomes in laboratory experiments[30,31].

SUPPLEMENTARY REFERENCES

1 Burkepile, D. E. *et al.* Nutrient supply from fishes facilitates macroalgae and suppresses corals in a Caribbean coral reef ecosystem. *Sci Rep* **3**, 1493, doi:10.1038/srep01493 (2013).

2 Briceno, H. O. B., J. N. . Annual report of the water quality monitoring project for the water quality protection program of the Florida Keys National Marine Sanctuary (Southeast Environmental Research Center, Florida International University, http://serc.fiu.edu/wqmnetwork/ 2012).

3 Paddack, M., Cowen, R. & Sponaugle, S. Grazing pressure of herbivorous coral reef fishes on low coral-cover reefs. *Coral Reefs* **25**, 461-472, doi:10.1007/s00338-006-0112-y (2006).

4 Schutte, V. G., Selig, E. R. & Bruno, J. F. Regional spatio-temporal trends in Caribbean coral reef benthic communities. *Mar Ecol Prog Ser* **402**, 115-122 (2010).

5 Maliao, R., Turingan, R. & Lin, J. Phase-shift in coral reef communities in the Florida Keys National Marine Sanctuary (FKNMS), USA. *Mar Biol* **154**, 841-853, doi:10.1007/s00227-008-0977-0 (2008).

6 Chiappone, M., Swanson, D. W. & Miller, S. L. Density, spatial distribution and size structure of sea urchins in Florida Keys coral reef and hard-bottom habitats. *Mar Ecol Prog Ser* **235**, 117-126 (2002).

7 Vega Thurber, R. *et al.* Chronic nutrient enrichment increases prevalence and severity of coral disease and bleaching *Global Change Biology* **20**, 544-554, doi:10.1111/gcb.12450 (2014).

8 Briceno, H. O. & Boyer, J. N. Annual report of the water quality monitoring project for the water quality protection program of the Florida Keys National Marine Sanctuary *Southeast Environmental Research Center, Florida International University*, 82 http://serc.fiu.edu/wqmnetwork/ (2012).

9 Dinsdale, E. A. *et al.* Microbial Ecology of Four Coral Atolls in the Northern Line Islands. *PloS one* **3**, -, doi:ARTN e1584
DOI 10.1371/journal.pone.0001584 (2008).

10 Burkepile, D. E. *et al.* Nutrient supply from fishes facilitates macroalgae and suppresses corals in a Caribbean coral reef ecosystem. *Scientific Reports* **3**, doi:10.1038/srep01493 (2013).

11 Newman, M. J. H., Paredes, G. A., Sala, E. & Jackson, J. B. C. Structure of Caribbean coral reef communities across a large gradient of fish biomass. *Ecology Letters* **9**, 1216-1227 (2006).

12 Gleason, D. Sedimentation and distributions of green and brown morphs of the Caribbean coral Porites astreoides Lamarck. *Journal of Experimental Marine Biology and Ecology* **230**, 73-89 (1998).

13 Jokiel, P. L. & Morrissey, J. I. Water motion on coral reefs - evaluation of the clod card technique. *Marine Ecology-Progress Series* **93**, 175-181 (1993).

14 Ferrari, R., Gonzalez-Rivero, M., Ortiz, J. C. & Mumby, P. J. Interaction of herbivory and seasonality on the dynamics of Caribbean macroalgae. *Coral Reefs* **31**, 683-692, doi:10.1007/s00338-012-0889-9 (2012).

15 Carpenter, R. C. Relationships between primary production and irradiance in coral reef algal communities. *Limnology and Oceanography* **30**, 784-793 (1985).
16 Hixon, M. A. & Bronstoff, W. N. Damselfish as keystone species in reverse: intermediate distrubance and diversity of reef algae. *Science* **220**, 511-513 (1983).
17 Smith, J., Smith, C. & Hunter, C. An experimental analysis of the effects of herbivory and nutrient enrichment on benthic community dynamics on a Hawaiian reef. *Coral Reefs* **19**, 332-342 (2001).
18 Burkepile, D. & Hay, M. Predator release of the gastropod Cyphoma gibbosum increases predation on gorgonian corals. *Oecologia* **154**, 167-173 (2007).
19 Miller, M. & Hay, M. Effects of fish predation and seaweed competition on the survival and growth of corals. *Oecologia* **113**, 231-238 (1998).
20 Miller, M. *et al.* Effects of nutrients versus herbivores on reef algae: A new method for manipulating nutrients on coral reefs. *Limnology and Oceanography* **44**, 1847-1861 (1999).
21 Birrell, C. L., McCook, L. J., Willis, B. L. & Diaz-Pulido, G. A. Effects of benthic algae on the replenishment of corals and the implications for the resilience of coral reefs. *Oceanography and Marine Biology: An Annual Review* **46**, 25-63 (2008).
22 Rasher, D. B. & Hay, M. E. Chemically rich seaweeds poison corals when not controled by herbivores. *Proceedings of the National Academy of Science* **107**, 9683-9688 (2010).
23 Vega Thurber, R. *et al.* Macroalgae Decrease Growth and Alter Microbial Community Structure of the Reef-Building Coral, Porites astreoides. *PloS one* **7**, e44246 (2012).
24 Smith, J. E. *et al.* Indirect effects of algae on coral: algae-mediated, microbe-induced coral mortality. *Ecology Letters* **9**, 835-845 (2006).
25 Morrow, K. M., Liles, M. R., Paul, V. J., Moss, A. & Chadwick, N. E. Bacterial shifts associated with coral–macroalgal competition in the Caribbean Sea. *Mar Ecol Prog Ser* **488**, 103-117 (2013).
26 Morrow, K. M., Paul, V. J., Liles, M. R. & Chadwick, N. E. Allelochemicals produced by Caribbean macroalgae and cyanobacteria have species-specific effects on reef coral microorganisms. *Coral Reefs* **30**, 309-320, doi:10.1007/s00338-011-0747-1 (2011).
27 Seymour, J. R., Simo, R., Ahmed, T. & Stocker, R. Chemoattraction to dimethylsulfoniopropionate throughout the marine microbial food web. *Science* **329**, 342-345, doi:10.1126/science.1188418 (2010).
28 Garren, M. *et al.* A bacterial pathogen uses dimethylsulfoniopropionate as a cue to target heat-stressed corals. *The ISME Journal* **8**, 999-1007, doi:10.1038/ismej.2013.210 (2013).
29 Thurber, R. V. *et al.* Metagenomic analysis of stressed coral holobionts. *Environmental Microbiology* **11**, 2148-2163 (2009).
30 Kuntz, N. M., Kline, D. I., Sandin, S. A. & Rohwer, F. Pathologies and mortality rates caused by organic carbon and nutrient stressors in three Caribbean coral species. *Mar Ecol Prog Ser* **294**, 173-180, doi:10.3354/meps294173 (2005).

31 Kline, D. I., Kuntz, N. M., Breitbart, M., Knowlton, N. & Rohwer, F. Role of elevated organic carbon levels and microbial activity in coral mortality. *Mar Ecol Prog Ser* **314**, 119-125 (2006).
